# Incorporating genetic similarity of auxiliary samples into eGene identification under the transfer learning framework

**DOI:** 10.1186/s12967-024-05053-6

**Published:** 2024-03-09

**Authors:** Shuo Zhang, Zhou Jiang, Ping Zeng

**Affiliations:** 1 Department of Biostatistics, School of Public Health, Xuzhou Medical University, Xuzhou, 221004 Jiangsu China; 2 Center for Medical Statistics and Data Analysis, Xuzhou Medical University, Xuzhou, 221004 Jiangsu China; 3 Key Laboratory of Human Genetics and Environmental Medicine, Xuzhou Medical University, Xuzhou, 221004 Jiangsu China; 4 Key Laboratory of Environment and Health, Xuzhou Medical University, Xuzhou, 221004 Jiangsu China; 5 Xuzhou Engineering Research Innovation Center of Biological Data Mining and Healthcare Transformation, Xuzhou Medical University, Xuzhou, 221004 Jiangsu China; 6 Jiangsu Engineering Research Center of Biological Data Mining and Healthcare Transformation, Xuzhou Medical University, Xuzhou, 221004 Jiangsu China

**Keywords:** Transfer learning framework, Joint effect test, Hierarchical modeling, Linear mixed model, Expression quantitative trait loci, Harmonic mean *P*-value

## Abstract

**Background:**

The term eGene has been applied to define a gene whose expression level is affected by at least one independent expression quantitative trait locus (eQTL). It is both theoretically and empirically important to identify eQTLs and eGenes in genomic studies. However, standard eGene detection methods generally focus on individual cis-variants and cannot efficiently leverage useful knowledge acquired from auxiliary samples into target studies.

**Methods:**

We propose a multilocus-based eGene identification method called TLegene by integrating shared genetic similarity information available from auxiliary studies under the statistical framework of transfer learning. We apply TLegene to eGene identification in ten TCGA cancers which have an explicit relevant tissue in the GTEx project, and learn genetic effect of variant in TCGA from GTEx. We also adopt TLegene to the Geuvadis project to evaluate its usefulness in non-cancer studies.

**Results:**

We observed substantial genetic effect correlation of cis-variants between TCGA and GTEx for a larger number of genes. Furthermore, consistent with the results of our simulations, we found that TLegene was more powerful than existing methods and thus identified 169 distinct candidate eGenes, which was much larger than the approach that did not consider knowledge transfer across target and auxiliary studies. Previous studies and functional enrichment analyses provided empirical evidence supporting the associations of discovered eGenes, and it also showed evidence of allelic heterogeneity of gene expression. Furthermore, TLegene identified more eGenes in Geuvadis and revealed that these eGenes were mainly enriched in cells EBV transformed lymphocytes tissue.

**Conclusion:**

Overall, TLegene represents a flexible and powerful statistical method for eGene identification through transfer learning of genetic similarity shared across auxiliary and target studies.

**Supplementary Information:**

The online version contains supplementary material available at 10.1186/s12967-024-05053-6.

## Background

In genomic studies the term “eGene” is used to define a gene whose expression level is affected by at least one independent single nucleotide polymorphism (SNP) nearby that gene (called cis-SNP); the corresponding SNP is called expression quantitative trait locus (eQTL) [1–4]. Identification of eGenes is imperative because genes are the major molecular unit in many biological processes, are interpretable and allow for subsequent network and pathway analyses [3]. The eQTL study provides not only the set of cis-variants related to gene expression, but also the set of eGenes for which eQTLs are identified [5–7], both of which exhibit an important implication regarding functional roles of significant loci discovered in genome-wide association studies (GWAS) in influencing diseases and intermediate phenotypes [8, 9].

Further, the finding that a trait-related SNP or gene detected in GWAS is also an eQTL or eGene renders substantial evidence for causality of this variant or gene. For example, in the GWAS of inflammatory bowel disease (IBD) [10], Repnik et al. [11] utilized eQTL mapping to analyze associated loci and confirmed several genes (e.g., *SLC22A5* and *ORMDL3*) involved in the pathogenesis of IBD. By integrating genetic associations from the GWAS of major depressive disorder (MDD) and brain eQTL data, Zhong et al. [12] discovered some risk variants contributed to MDD susceptibility through affecting the expression of *FLOT1*, providing new insights into the etiology of this disorder.

The standard method of identifying eGene is to examine the association of cis-SNPs of a given gene with its expression level to assess whether any of them are significant. The permutation-based multiple testing correction is required to properly account for the linkage disequilibrium (LD) among variants, which is computationally intensive although novel improvements have been recently proposed [3, 13]. Due to the issue of multiple comparisons, a relatively small *P* value might not be sufficiently small to reach the significance level. Thus, the standard analysis is often underpowered for eGene detection [2, 14].

Alternatively, we consider the discovery of eGene from a statistical perspective of variant-set association analysis by evaluating the joint influence of all cis-SNPs on gene expression. SNP-set analysis has been widely employed in genomic studies [15–17], where a set of variants defined a priori within a gene or other genetic unit are analyzed collectively to examine their joint effects on a disease or phenotype, so that the power of eGene detection is acceptable even individual eQTLs cannot be detected. Therefore, compared to the standard analysis above, the SNP-set based method is expected to be more powerful to identify eGene because it aggregates multiple weakly correlated signals and reduces the burden of multiple comparisons [15].

However, the current SNP-set based approach is likely still sub-optimal if there exists additional knowledge that is informative for eGene discovery in target samples. For example, eQTLs are shared across tissues [18, 19], it is feasible to incorporate such sharing to boost power for association analysis [20–22] or improve accuracy for gene expression prediction in a specific tissue [23, 24]. In addition, it has been demonstrated that leveraging functional genomic annotations of variants (e.g., distance from transcription start site or certain histone modification) or utilizing cross-tissue genetic similarity can also increase power of eGene and eQTL detection [1, 2].

To integrate genetic information available from external studies, we here propose a multilocus-based eGene identification method by borrowing the idea of transfer learning [25–29] as well as the idea of SNP-set association analysis [15–17]. Particularly, within the transfer learning framework, we refer to individuals under analysis as target samples, and individuals of different but closely related studies as auxiliary samples. For efficient knowledge transferring, we assume the effect of cis-SNP in the target study is analogous to that in auxiliary studies, and suppose the former could be predictive by the cis-SNP effect of auxiliary samples. Consequently, our eGene identification consists of two components: the first component represents the indirect influence of auxiliary study after transfer learning, and the second component represents the direct effect of target study. We refer to the proposed eGene identification statistical framework as TLegene. Further, even no eQTLs are discovered in target samples, significant eGenes are still likely identified due to the indirect influence of auxiliary samples. If the target samples are strongly associated to the auxiliary samples, these identified eGenes are likely biologically meaningful.

Specifically, to implement our method, we first make a novel decorrelation modification to generate two independent statistics for each of the two components [20]; then we can easily construct a unified joint test based on the two uncorrected statistics through various combination strategies including optimal weighted linear combination (TLegene-oScore), adaptive weighted linear combination (TLegene-aScore), and Fisher’s combination (TLegene-fScore). To further enhance power, we employ the recently developed harmonic mean *P*-value method (TLegene-HMP) [30] to aggregate the strength of the three joint test methods. Finally, we apply TLegene to eGene identification for ten TCGA cancers (Table 1) which have explicit relevant tissues available from the GTEx project [18], and learn genetic effect of SNP in TCGA from GTEx. We also adopt TLegene to the Geuvadis project [31] to further evaluate its usefulness in non-cancer studies. Overall, in line with the simulations, we identified more candidate eGenes with TLegene than the methods that did not consider knowledge transfer across target and auxiliary studies, and demonstrated that TLegene was powerful in real-data applications.

**Table 1:** Descriptive statistics of the ten TCGA cancers after combining GTEx

Cancer	*n*_0_	*n*_1_	*m*_0_	*m*_1_	Age	Female/male	Stage/grade (1/2/3/4/5)	Tissue in GTEx	*n*_2_	*m*_2_	*k*_0_	*k*_1_
ACC	97	75	9,473,821	4,592,516	47.6 ± 16.5	50/25	8/33/16/18/0	Adrenal gland	175	8,886,529	7371	6897
BRCA	1283	736	10,639,477	2,211,750	58.8 ± 13.0	736/0	138/408/174/11/5	Breast mammary	251	8,886,322	7714	4236
COAD	570	201	16,825,606	3,873,035	66.0 ± 13.0	97/104	34/78/62/27/0	Colon transverse	246	8,879,795	7716	6221
LIHC	469	166	12,269,510	3,359,173	62.7 ± 14.1	73/93	79/44/39/4/0	Liver	153	8,871,933	7143	5318
LUAD	577	384	15,199,217	3,607,888	65.9 ± 9.9	213/171	216/90/62/16/0	Lung	383	8,853,514	7781	666
LUSC	765	344	11,830,021	3,130,896	67.1 ± 8.8	94/250	176/117/48/3/0	Lung	383	8,835,520	7781	5464
OV	758	455	5,635,755	1,373,814	60.2 ± 11.4	455/0	9/19/353/74/0	Ovary	122	8,798,367	7499	410
PAAD	223	159	14,099,808	4,410,547	65.6 ± 10.8	69/90	20/130/4/5/0	Pancreas	220	8,764,053	7237	6709
STAD	544	249	12,286,585	3,183,096	64.8 ± 10.2	97/152	33/75/128/13/0	Stomach	237	8,700,105	7586	5482
UCEC	605	368	17,131,516	3,747,929	64.4 ± 10.8	368/0	239/33/79/17/0	Uterus	101	8,886,529	7532	6642

*n*_0_: the initial sample size in TCGA; *n*_1_: the sample size after quality control;* n*_2_: the sample size in GTEx;* m*_0_: the initial number of SNPs in TCGA; *m*_1_: the number of shared SNPs between TCGA and GTEx;* m*_2_: the initial number of SNPs in GTEx; *k*_0_: the number of genes after combination; *k*_1_: the number of genes after quality control; ACC: adrenocortical cancer; BRCA: breast cancer; COAD: colon cancer; LIHC: liver cancer; LUAD: lung adenocarcinoma; LUSC: lung squamous cell carcinoma; OV: ovarian cancer; PAAD: pancreatic cancer; STAD: stomach cancer; UCEC: endometrioid cancer

## Methods

### SNP-set based eGene identification within linear mixed model

In TLegene we analyze one gene at each time. Suppose that there are *n* individuals and *m* cis-SNPs denoted by **G** = (*g*_1_, …, *g*_*m*_) for a given gene in the target study; we aim to assess whether the expression level (denoted by ***e***) of a specific gene is affected by its local variants. To examine such relation, we construct a linear mixed model [32, 33]

1 $${\varvec{e}} = {\mathbf{X}}{\varvec{\alpha}}{ + }{\mathbf{G}}{\varvec{\beta}}{ + }{\varvec{\varepsilon}},$$

where **X** stands for the design matrix of *p* covariates, with ***α*** = (*α*_1_, …, *α*_*p*_) the fixed effect vector; ***β*** = (*β*_1_, …, *β*_*m*_) is the random effect vector for these SNPs, *β*_*j*_ ~ *N*(0, τ) (*j* = 1, …, *m*); and ***ε*** = (*ε*_1_, …, *ε*_*n*_) is the normal residual vector. Under this modeling specification, evaluating whether the focused gene is an eGene is equivalent to examining the null hypothesis *H*_0_: τ = 0. The variance component-based score test (denoted by Score) is often employed as it is powerful across distinct settings [15, 20, 34].

### Transfer learning via genetic similarity integration

To make efficient use of existing auxiliary data resources that are closely analogous to the target samples, we precede TLegene using transfer learning techniques [35–37]. First, let **γ** = (γ_1_, …, γ_*m*_) be the vector of known SNP effects obtained from summary statistics of auxiliary studies; then, we assume the genetic effect *β* in the target study can be predicted by **γ** in a given auxiliary study [20]

2 $$\beta_{j} = {\upgamma }_{j} \times \theta + b_{j} ,j = 1,..,m,$$

where *θ* is the indirect effect of auxiliary study, and *b*_*j*_ (*j* = 1, …, *m*) is the direct effect not completely interpreted by auxiliary data. We still assume *b*_*j*_ ~ *N*(0, τ). Here, we are attempting to learn *β* based on auxiliary effect estimate. Finally, plugging (2) into (1), we obtain the TLegene model

3 $$\begin{aligned} {\varvec{e}} & = {\mathbf{X}}{\varvec{\alpha}}{ + }{\mathbf{G}}{(}{{\varvec{\upgamma}}} \times \theta { + }{\varvec{b}}{) + }{\varvec{\varepsilon}}, \, b_{j} \sim N(0,{\uptau }) \\ & = {\mathbf{X}}{\varvec{\alpha}}{ + (}{\mathbf{G\upgamma }}{)} \times \theta { + }{\mathbf{G}}{\varvec{b}}{ + }{\varvec{\varepsilon}} \\ \end{aligned}$$

where **Gγ** is a weighted genetic score which is also called burden component [38, 39], with *θ* quantifying its association with the expression level. Under the TLegene modeling framework, the null hypothesis turns into

4 $$H_{0} :\theta = 0\;{\text{and}}\;{\varvec{b}} = 0 \Leftrightarrow H_{0} :\theta = 0\;{\text{and}}\;{\uptau } = 0.$$

This is a joint test which requires simultaneously assessing the significance of both fixed effects and random effects: the first part of *H*_0_ evaluates the indirect influence of auxiliary samples, whereas the second part assesses the direct impact of target samples. If *θ* = 0, model (3) reduces to $\user2{e } = {\mathbf{X}}{\varvec{\alpha}} + {\mathbf{G}}{\varvec{b}} + {\varvec{\varepsilon}},$ testing the effects of all cis-SNPs equal to zero (*H*_0_: ***b*** = 0) is equivalent to examining the variance of ***b*** equal to zero (*H*_0_: τ = 0), which is a special case of the joint hypothesis test given in (4), and is particularly powerful when only the target effects are present [40]. The code for implementing the hypothesis test of TLegene is freely available at https://github.com/biostatpzeng/TLegene.

### Joint test in TLegene

#### Combination of two independent score tests

We here employ the score test to examine the joint null hypothesis given in because this test method successfully avoids estimating the variance parameter under the alternative and is thus computationally efficient. We can easily obtain the respective score statistics for *θ* and τ under the null, with the score statistic for *θ* following a χ^2^ distribution with one degree of freedom and the score statistic for τ following a mixture of χ^2^ distribution [40]; however, the two statistics are statistically correlated if there are no any additional specifications [20–22]. Therefore, it is not straightforward to derive their joint null distribution.

To overcome this challenge, we decorrelate the two score statistics so that they could be asymptotically independent. Specifically, we first derive the score statistic of *θ* under *H*_0_ (i.e., *θ* = 0 and τ = 0) as usual, but we next derive the score statistic of τ under the null of only τ = 0 without restricting *θ* = 0. By doing this, it guarantees that the two score statistics are uncorrelated [20–22]. The decorrelation greatly simplifies the derivation of joint test statistic, and independence itself offers various possibilities of aggregating the two test statistics so that we can maximize the ability to target different types of alternatives. Finally, we construct the joint test statistic by combining the two unrelated statistics through several combination methods such as optimally weighted linear combination (TLegene-oScore), adaptively weighted linear combination (TLegene-aScore), and Fisher’s combination (TLegene-fScore) [20–22]; technical details of the three combination methods are given in Additional file 1.

#### Aggregation of three combination-based joint test methods

The three combination-based joint test methods (i.e., TLegene-oScore, TLegene-aScore, and TLegene-fScore) have distinct advantages and would show higher power under respective modeling assumptions. To circumvent the difficulty of selecting an optimal one, we aggregate their strengths via the recently developed harmonic mean *P*-value method (TLegene-HMP) to generate an omnibus test [30]

5 $$T = {1 \mathord{\left/ {\vphantom {1 {\left( {\sum\limits_{t = 1}^{3} {\frac{1}{{P_{t} }}} } \right)}}} \right. \kern-0pt} {\left( {\sum\limits_{t = 1}^{3} {\frac{1}{{P_{t} }}} } \right)}},P = \int_{\frac{1}{T}}^{\infty } {f_{x} \left( {x|\log T + 0.{874},\frac{\pi }{2}} \right)dx}$$

where *P*_*t*_ (*t* = 1, 2, 3) denotes the *P* value yielded from each of these methods, and *f*_*x*_ denotes the Landau distribution probability density function. It has been demonstrated that HMP is robust against positive dependency among combined *P* values [30, 41].

### Simulations for type I error control and power evaluation

We now carried out simulation studies to evaluate the performance of type I error control and power of TLegene. To this aim, we extracted common SNPs within an LD block of genotypes from the 1000 Genomes Project (*n* = 503) [42] and the Geuvadis project (*n* = 465) [31]. We randomly selected *m* SNPs (with *m* following a uniform distribution from 20 to 50) and 165 individuals from the 1000 Genomes Project to generate gene expression in the auxiliary study; among these selected SNPs, 30%, 50% or 70% were null, while the remaining had non-zero effects following a normal distribution with a mean zero and a particular variance so that the gene expression phenotypic variance explained (PVE) by SNPs would be 30% or 50%.

Second, in the target study we created gene expression using 300 individuals randomly selected from the Geuvadis project with the same set of selected SNPs, but calculated the effect as *β* = *γ* × *θ* + *b*, with *b* having a normal distribution with a mean zero and a variance τ. Two independent covariates were also generated (i.e., *X*_1_ is binary and *X*_2_ is continuous) in both target and auxiliary samples, each having an effect of 0.50. To evaluate type I error control, we set *θ* = 0 and τ = 0 with 10^5^ replications. To evaluate power, we specified *θ* = 0, 0.1, 0.2, 0.3 or 0.4, and *τ* = 0, 0.02 or 0.04 (with at least one of *θ* and τ being non-zero) with 10^3^ replications.

To assess the performance of power of TLegene when there existed obvious differences in the sample sizes of the auxiliary and target studies, we performed our simulations with 400 randomly selected individuals from the 1000 Genomes Project as the auxiliary samples and 100 randomly selected individuals from the Geuvadis project as the target samples. We also conducted our simulations with 100 randomly selected individuals from the 1000 Genomes Project as the auxiliary samples and 400 randomly selected individuals from the Geuvadis project as the target samples. Other simulation settings were analogous to those as done before, but only *θ* = 0.3 or/and τ = 0.04 were considered.

### Real data applications

#### TCGA datasets and quality control

We applied TLegene to multiple TCGA cancers to identify eGenes in various tumor tissues. We only focused on cancers for which there existed an explicit tissue available from GTEx [18]; thus, TCGA was our target study and GTEx was our auxiliary study. To avoid the influence of ethnic heterogeneity, we only contained patients of European ancestry. We selected several clinical covariates such as age, gender as well as the tumor pathological stage (Table 1), because these variables could be obtained for the majority of TCGA patients [20, 43–45]. We would choose the clinical stage when the tumor pathological stage was unavailable for some tumors (e.g., OV). For each cancer we only retained samples of primary tumor tissues, and imputed missing values via the multivariate imputation by chained equation method. For genotypes of each tumor in TCGA, we performed quality control and imputation, with the details described elsewhere [20, 46]. The sample size ranged from 75 for ACC to 455 for OV, and the number of SNPs ranged from 1,373,814 for OV to 4,592,516 for ACC.

#### GTEx summary statistics and the alignment with TCGA

For each cancer, we obtained summary statistics data of the related tissue from GTEx (version 7) [18]. The sample size ranged from 101 for uterus to 383 for lung, and the number of SNPs ranged from 8,700,105 for liver to 8,886,529 for adrenal gland. Then, we carried out stringent quality control (Table 1): (i) reserved SNPs with MAF > 0.05; (ii) excluded non-biallelic SNPs and those with strand-ambiguous alleles; (iii) excluded SNPs without rs ID or removed duplicated variants; (iv) removed SNPs not in the TCGA; (v) removed SNPs whose alleles did not match those in TCGA; (vi) aligned the effect allele of SNP between TCGA and GTEx. Finally, the sample size ranged from 75 for ACC to 455 for OV, the number of shared SNPs ranged from 1,373,814 for OV to 4,592,516 for ACC, and the final number of genes included ranged from 4236 for BRCA to 6897 for ACC. The details of used datasets are described in Table 1.

#### Correlation of SNP effects of SNPs between GTEx and TCGA

To get an initial insight of the correlation of two types of SNP effects between TCGA and GTEx, we first generated the marginal effect of SNP in TCGA through a single-marker linear model by regressing the expression of every gene on each of its cis-SNP in TCGA while adjusting for cancer-specific covariates such as age and tumor stage [14]. Then, we performed a linear regression for SNP effects between TCGA and GTEx to characterize their relation. For numerical stability, we only focused on genes with at least five SNPs.

#### Traditional method of identifying eGene

For every eGene discovered by TLegene across the cancers, we also performed the traditional linear regression for eGene identification by examining the association of each cis-SNP with the expression level. We adjusted for the same covariates as those in TLegene and applied Bonferroni’s method to explain the multiple test issue.

#### Geuvadis project

We further applied TLegene to the Geuvadis project [31] to identify eGenes in non-cancer studies. The Geuvadis project contains gene expression measurements in lymphoblastoid cell lines for 465 individuals. Following previous work [32, 47, 48], we mainly analyzed protein-coding genes and lincRNAs defined according to GENCODE (version 12) [49]. We removed zero-count low-expressed genes in at least half of the individuals, obtaining 15,810 genes. Then, in terms of the previous study [50], we performed PEER normalization to remove confounding effects and unwanted variation. All individuals in Geuvadis were sequenced for their genotypes in the 1000 Genomes Project. Here, the Geuvadis individuals were our target samples, and the individuals with the cells EBV transformed lymphocytes tissue from the GTEx project were our auxiliary samples. A total of 7269 genes and the number of 3,124,631 shared SNPs were finally included.

## Results

### Type I error control and power evaluation

**Fig. 1:**
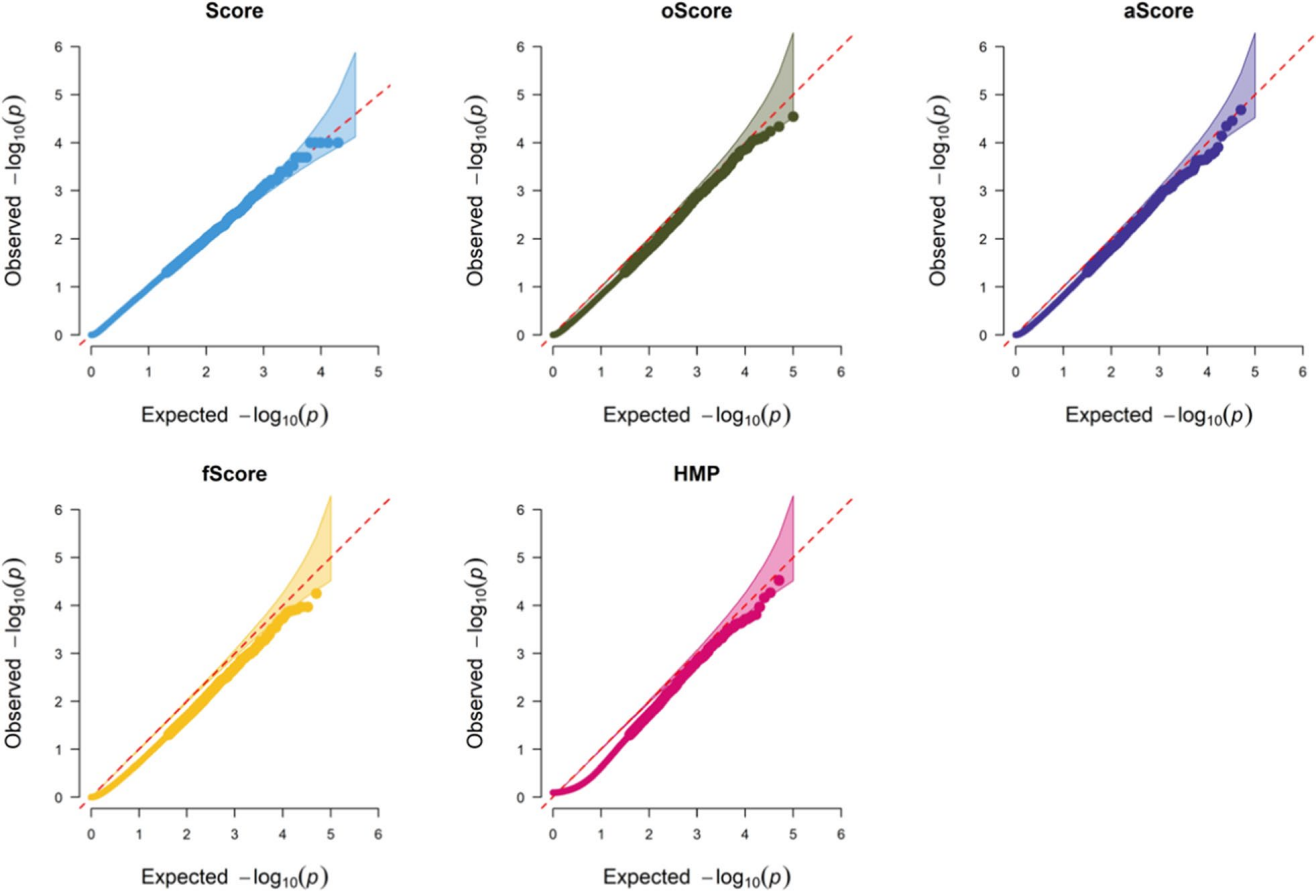
QQ plots evaluating the performance of type I error control for Score, TLegene-oScore, TLegene-aScore, TLegene-fScore, and TLegene-HMP under the null in which both *θ* and τ were zero

First, we showed that all tests, including Score (i.e., the variance-component based score test), TLegene-oScore, TLegene-aScore, TLegene-fScore and TLegene-HMP, could maintain correct control of type I error (Fig. 1). We next compared the power of these tests under distinct alternative scenarios. To save space, here we only presented the estimated power under the scenario where the PVE in the auxiliary study was set to 0.3 or 0.5, *θ* (the effect of the auxiliary study) was set to 0 or 0.1, and τ (the variance of the direct effect of target study) was set to 0 or 0.02. The results for other scenarios were displayed in Additional file 1: Figs. S1–S9.

**Fig. 2:**
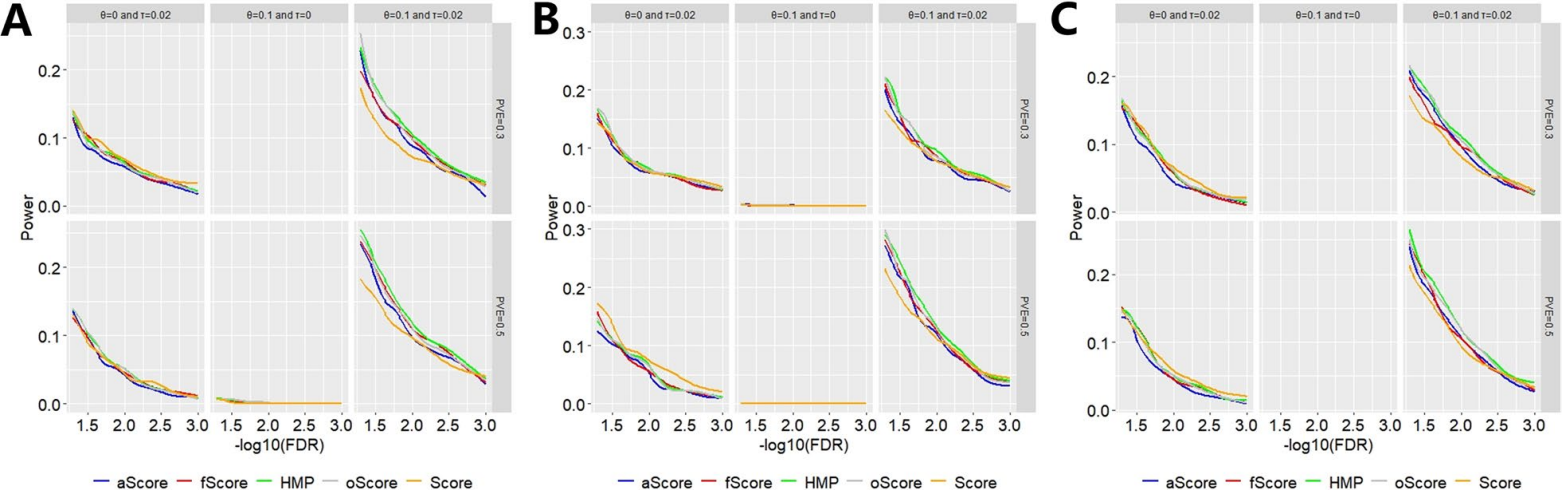
Comparison of power for the five test methods under the alternative scenarios. Here, the PVE in the auxiliary study was set to 0.3 (top) or 0.5 (bottom), the sample size of the target study was 300 and the sample size of the auxiliary study was 165, *θ* = 0.1 or/and τ = 0.02. **A** 30% of SNPs were null; **B** 50% of SNPs were null; **C** 70% of SNPs were null

As shown in Fig. 2, when the sample size of the target study was 300 and the sample size of the auxiliary study was 165, we found that Score was powerful when only the target effect was present (e.g., *θ* = 0 and τ = 0.02), but suffered from power reduction when only the indirect auxiliary impact existed (e.g., *θ* = 0.4 and τ = 0) (Additional file 1: Fig. S6). In contrast, compared to Score, the three joint tests (i.e., TLegene-oScore, TLegene-aScore and TLegene-fScore) and the omnibus test (i.e., TLegene-HMP) were better when both the target and auxiliary effects existed (e.g., *θ* = 0.3 and τ = 0.04) (Additional file 1: Fig. S7). Moreover, across all scenarios of power evaluation, TLegene-HMP was more powerful or comparable compared to other methods.

When the sample sizes of the target and auxiliary studies were 100 and 400, respectively, we observed that the three combined tests and TLegene-HMP were better compared to Score when both the target and auxiliary influences existed (Additional file 1: Fig. S8). However, when the sample size of the auxiliary study was 100 and the sample size of the target study was 400, we did not observe that TLegene showed a substantially higher power compared to Score. This was likely due to the small sample size of the auxiliary study (Additional file 1: Fig. S9), which implied little learnable information from auxiliary samples to target samples and resulted in high uncertainty of auxiliary genetic effects during transfer learning.

### Correlation of cis-SNP effects for each gene between TCGA and GTEx

In the real application, we first assessed the relation of cis-SNP effects for all genes between TCGA and GTEx, with the estimated correlation summarized in Table 2. We found that the effects of the two sets of SNPs were substantially dependent for a great number of genes in each cancer. On average, most of genes (~ 75.4%) (ranging from 72.0% for OV to 77.5% for LUAD) had a significant regression coefficient (false discover rate [FDR] < 0.05), and a small proportion of genes (~ 5.2%) had a coefficient of determination (*R*^2^) larger than 0.10, suggesting the SNP effects in GTEx did possess the ability to inform the SNP effects for some genes in TCGA.

**Table 2:** Summary information of cis-SNPs for the ten cancers and the correlation of cis-SNP effects for each gene in TCGA and GTEx

Cancer	Median	*M* (%)	*R*^2^ > 0.10 (%)
ACC	3096	5227 (75.5)	237 (3.4)
BRCA	2878	3191 (75.3)	265 (6.3)
COAD	3478	4724 (69.8)	283 (4.2)
LIHC	3119	4018 (73.5)	191 (3.5)
LUAD	3534	4701 (77.5)	232 (3.8)
LUSC	3313	4149 (75.9)	289 (5.3)
OV	1571	3176 (70.9)	766 (17.1)
PAAD	3560	5067 (75.5)	203 (3.0)
STAD	3397	4176 (76.2)	174 (3.2)
UCEC	3324	4663 (73.8)	137 (3.4)

Median: the median number of cis-SNPs across genes; *R*^2^: the determination coefficient of the cis-SNPs effects for each gene in the linear regression; *M*: the number of genes whose regression coefficient is significant (FDR < 0.05)

**Fig. 3:**
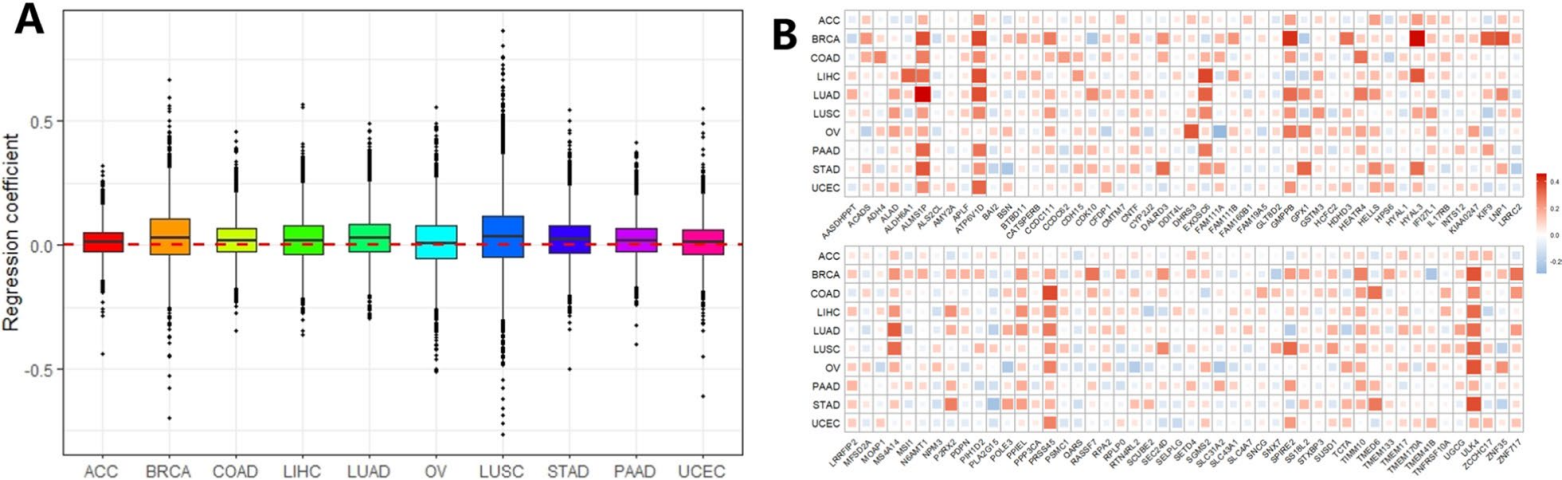
**A** Distribution of regression coefficient for each gene when regressing the SNP effect in TCGA on that in GTEx. **B** Summary of 96 genes whose estimated regression coefficients simultaneously significant (FDR < 0.05) across all cancers; the magnitude of the regression coefficient is represented by the density and size of the color

In addition, we recognized that the regression coefficients were positive for some cancers but negative for other cancers (Fig. 3A), indicating the distinct influence of SNPs on the regulation of gene expression. Particularly, there were 96 genes whose regression coefficients were significant across all the ten cancers in TCGA (Fig. 3B). In short, the relatively high correlation of SNP effects between TCGA and GTEx demonstrated substantial similarity in expression regulation. Therefore, it was worthwhile to learn SNP effects of TCGA through GTEx to improve power for eGene identification.

### Correlation evaluation in TCGA cancer

As described above, the effects of SNPs in GTEx were predictive and informative for the genetic influence of variants in TCGA; we could thus reasonably assume that smaller *P* values would be generated when implementing TLegene. Consequently, we expected higher detection rate of eGenes (*P* < 0.05) for specific genes with significant regression coefficients compared to those with insignificant ones. To validate this conjecture, for each cancer we classified all genes into four different groups by regression coefficients (whether FDR < 0.05) and the association results of the four tests constructed in TLegene (whether *P* < 0.05) (Additional file 1: Tables S1–S4).

**Fig. 4:**
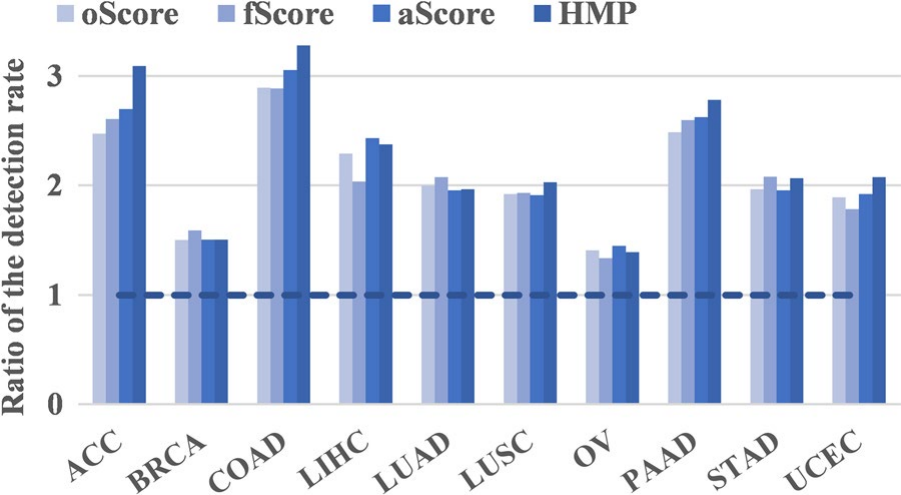
Improvement in detection rate for genes with significant regression coefficients for all the ten TCGA cancers after learning SNP effect of TCGA from those of GTEx. The improvement was calculated by the ratio of the detection rate of genes with significant regression coefficients to the detection rate of genes with non-significant regression coefficients; thus, a ratio > 1 indicates an increase in detection rate

Taking COAD as an example, there were 4724 (= 602 + 4211) genes with significant regression coefficients (FDR < 0.05), whereas 1497 (= 66 + 1431) with non-significant regression coefficients (FDR > 0.05); of these genes, the *P* values of 602 (12.7% = 602/4724) and 66 (4.4% = 66/1497) genes in TLegene-oScore were less than 0.05, indicating that TLegene-oScore had an approximate three-fold higher likelihood (2.9 = 12.7/4.4) of identifying an eGene after informative transfer learning. We further employed the χ^2^ test to formally evaluate the difference in detection rate (e.g., 12.7% vs. 4.4%), and observed significant evidence in detection rate for nearly all cancers in TCGA and significant increase in detection rate for TLegene (Fig. 4).

### Discovered eGenes and their characteristics

#### Detected eGenes by TLegene

The number of eGenes discovered by TLegene is summarized in Table 3. Particularly, among these cancers only few eGenes (Bonferroni-corrected *P* < 0.05) were discovered for ACC, OV, and UCEC. Totally, 169 distinct eGenes were identified, of which 88 were identified only for one cancer, while 81 eGenes were detected for at least one type of ten TCGA cancers (Additional file 1: Table S5). Specifically, one eGene was shared by eight cancers, three eGenes were shared by seven cancers, five eGenes were shared by six cancers, six eGenes were shared by five cancers, seven eGenes were shared by four cancers, 20 eGenes were shared by three cancers, and 39 eGenes were shared by two cancers (Additional file 1: Fig. S10). Furthermore, we found that TLegene-HMP identified 325 eGenes across all the ten TCGA cancers, with more discoveries compared to the other TLegene tests. We also performed Score [51, 52], but failed to detect any eGenes.

**Table 3:** Number of significant genes identified by TLegene for all the ten TCGA cancers

Cancer	TLegene				*G*_1_	*G*_2_	Linear regression (%)	PancanQTL (%)
	oScore	aScore	fScore	HMP				
ACC	2	2	2	2	2	2	2 (100)	2 (100)
BRCA	72	73	85	83	68	87	74 (85.1)	80 (92.0)
COAD	39	40	35	39	34	41	34 (82.9)	36 (87.8)
LIHC	13	13	9	12	9	13	8 (61.5)	13 (100)
LUAD	68	67	65	69	60	71	54 (76.1)	69 (97.2)
LUSC	39	40	40	40	37	42	32 (76.2)	40 (95.2)
OV	9	9	10	10	9	10	7 (70.0)	10 (100)
PAAD	42	43	40	41	36	47	38 (80.9)	41 (87.2)
STAD	21	22	24	24	21	24	20 (83.3)	22 (91.7)
UCEC	5	5	4	5	3	6	5 (83.3)	4 (66.7)
Total	310	314	314	325	279	343	274 (79.9)	317 (92.4)

*G*_1_: the number of eGenes simultaneously identified by all the four TLegene tests;* G*_2_: the number of eGenes identified by any of the four tests; the last two columns denote the number (proportion) of eGenes replicated by the traditional eGene identification method using linear regression or PancanQTL

For the identified eGenes of each cancer, we carried out the traditional method for eGene discovery via linear regression to identify the association of each cis-SNP in a given gene with its expression level. The results showed that on average 79.9% of the eGenes identified by TLegene could be replicated by the traditional approach (Table 3 and Additional file 1: Fig. S11).

We also tried to validate the eGenes discovered by TLegene (*G*_*2*_ in Table 2) with those identified eGenes in PancanQTL [53]. PancanQTL aimed to comprehensively provide cis-eQTLs and trans-eQTLs in 33 cancer types from TCGA, which allowed us to obtain validated eGenes (http://gong_lab.hzau.edu.cn/PancanQTL/). We found that that on average 92.4% of the eGenes identified by TLegene could be repeated by PancanQTL (Table 3 and Additional file 1: Fig. S11).

We further selected only significant cis-SNPs (FDR < 0.05) in explicit tissues available from GTEx to estimate the correlation of SNP effects between TCGA and GTEx for these identified eGenes for each cancer. The results showed that, except a few eGenes, *R*^2^ of eGenes estimated only with significant cis-SNPs were much higher than that of eGenes estimated with insignificant cis-SNPs. Taking ACC as an example, two eGenes (*ERAP2* and *LRRC37A2*) were identified, the *R*^2^ was 0.3 or 0.4 estimated with only significant cis-SNPs, respectively, which was larger than the value (0.1 or 0.2) estimated with insignificant cis-SNPs (Additional file 1: Fig. S12).

#### Characteristics of eGenes and functional enrichment analysis

Previous studies provided evidence for some of these identified eGenes to support their connections with certain cancers, with some examples given in Additional file 1. Moreover, we performed KEGG and GO enrichment analyses for these eGenes using the clusterProfiler package [54] (Additional file 1), and identified several enriched pathways for BRCA, LUAD, LUSC, PAAD and STAD (Additional file 1: Figs. S13–S14). We also conducted functional analysis with FUMA [55]. However, except for the eGenes found in BRCA, which were enriched in whole blood tissue, as well as the eGenes detected in STAD, which were enriched in brain putamen basal ganglia tissue, we did not find that the expression levels of these identified genes were significantly enriched in the most relevant tissue for the corresponding cancer (Additional file 1: Figs. S15–S16); see Additional file 1 for more information.

### Applying TLegene to Geuvadis

When applying TLegene to the Geuvadis data, we found that TLegene-HMP identified 329 eGenes, which was slightly less than the number of eGenes identified by TLegene-fScore (340), but was much more than the number of eGenes identified by TLegene-oScore (221) and TLegene-aScore (288). We also performed the score test, but only identified 27 eGenes. Furthermore, the KEGG and GO enrichment analyses and the functional analysis with FUMA [55] identified several enrichment pathways (Additional file 1: Fig. S17), and showed these eGenes were mainly enriched in cells EBV transformed lymphocytes tissue (Additional file 1: Fig. S18).

## Discussions

### Summary of our proposed method and real data applications

In this paper, we have proposed a powerful eGene identification method called TLegene by efficiently transferring auxiliary information into target task [35–37]. The efficient utilization of existing eQTL knowledge acquired from different but related problems can not only enhance power but also save time and cost for additional data collection. We derived two separate score test statistics for the auxiliary effect and the target effect, respectively, and carried out novel combination to construct three joint tests in TLegene; however, it is hard to know which of them are optimal in practice. We thus sought to further aggregate the advantages of these joint tests. The challenge emerged due to the non-negligible positive dependence among test statistics of the three methods because they were implemented on the same dataset with the similar logic [20, 30, 41]. The minimum *P*-value and permutation methods can be used but they are either computationally intensive or difficult to conduct since the correlation structure is unknown. To overcome this difficulty, we employed HMP (i.e., TLegene-HMP) to generate an omnibus test. The advantage of HMP is that it allows us to aggregate correlated *P*-values obtained from distinct tests into a single well-calibrated *P*-value without the knowledge of correlation structure while maintaining correct control of type I error [30, 41].

In our real application, by examining the genetic effects of SNP between GTEx and TCGA, we observed substantial similarity between the two types of impacts for many genes. This offered pivotal foundation for transferring knowledge acquired from GTEx to TCGA for power improvement when identifying eGenes. As expected, we revealed that leveraging this similarity could identify substantially more eGenes which were otherwise not detected if not transferring such knowledge. We also applied TLegene to the Geuvadis project, and showed that TLegene identified more eGenes and that these eGenes were mainly enriched in cells EBV transformed lymphocytes tissue, which further demonstrated the usefulness of TLegene in non-cancer studies.

However, we noted that many of eGenes identified by TLegene in the TCGA project were not enriched in the same cancer tissue. We here discussed this point from multiple aspects. First, it is important to highlight that we have shown supportive evidence for these discovered relationships between the identified eGenes and TCGA cancers (Additional file 1), and most of the eGenes could be replicated by the traditional method or PancanQTL [53]. Furthermore, we found that some eGenes not replicated by PancanQTL had been demonstrated to be likely associated with certain cancers. For example, *HLA-L* showed strong evidence of association with lung cancer [56], *PSORS1C1* was implicated in adenocarcinoma at the gastroesophageal junction [57]. Therefore, these eGenes were still of great value for further cancer research.

Second, when applying TLegene to the Geuvadis data, we found that TLegene-HMP identified 329 eGenes and that these eGenes were mainly enriched in cells EBV transformed lymphocytes tissue, which demonstrated the usefulness of TLegene in non-cancer studies. Moreover, compared to the number of eGenes identified in Geuvadis, the number of eGenes identified in TCGA was much less, which implied that more TCGA eGenes were not discovered yet and may be a possible reason why the identified eGenes in TCGA were not enriched in cancer-specific tissues.

Third, eGenes likely play functional roles in only a small fraction of biological processes and pathways involved in cancer development and progression [58, 59], and may be also involved in other processes or pathways that are not directly related to cancers. For examine, the discovered eGenes may be associated with normal tissue development, cell homeostasis, or other biological functions that indirectly promote cancer susceptibility or progress [60, 61]. Therefore, the lack of enrichment in cancer-associated tissues does not negate the potential involvement of eGenes in the cancer biology.

### Comparison with previous work

TLegene distinguishes itself from previous approaches in four aspects. First, TLegene is based on a set of cis-SNPs rather than individual variants. This SNP-set based method has been shown to possess higher power in many cases compared to single-marker analysis [15].

Second, TLegene is different from Func-eGene [2] which attempted to improve the power of eGene detecting with own genomic functional annotations of variants rather than auxiliary information. In addition, Func-eGene analyzed individual cis-variants and was time-consuming; thus, it was not yet applicable for simultaneously handling a great many annotations. In contrast, integrating multiple auxiliary studies into TLegene is conceptually and practically easy.

Third, from the statistical and methodological perspective, TLegene is related to RECOV [1]. However, RECOV was proposed to utilize information shared cross distinct tissues with the aim to identify eGene within any of tissues under consideration, which is different from our objective that we hope to discover eGene within one special target tissue by exploiting knowledge acquired from different but possibly related auxiliary samples. Moreover, RECOV also focused on eGene identification with individual variants.

Fourth, the most pronounced distinction is that, as far as we know, TLegene is among the first to explicitly construct the eGene identification method under the transfer learning framework [25–29, 35–37]. Actually, the pervasive genetic similarity of shared across distinct tissues [18], populations [62–69] and studies [70–78] provides solid foundation for eGene identification by exploiting existing genomic knowledge via transfer learning.

Furthermore, from a Bayesian point of view [79], the genetic overlap between the target and auxiliary studies was integrated via a prior function in TLegene. The effect similarity of many genes means informative and predictive, which would produce more accurate parameter estimation and more powerful hypothesis test method. Compared to the classical Bayesian model, the main difference of TLegene is that we adopted a probabilistic approach [80], instead of sampling techniques or variational methods [33], to estimate unknown parameters and assess the hypothesis test. In addition, in TLegene we ignored the uncertainty of the SNP effects estimated from auxiliary populations.

## Future study directions

Out proposed method is not without limitations. Obviously, TLegene did not consider how to further pinpoint eQTLs within a discovered eGene. The step-down procedure can be applied to determine which cis-variants likely drive the association [81]. Indeed, using the step-down procedure after detecting eGenes, we observed that approximately 90% of discovered eGenes had only one independent eQTL and the remaining had at least two uncorrelated eQTLs, indicating allelic heterogeneity of gene expression [82]. However, the step-down procedure is not suitable for eGene if the gene only includes cis-SNPs with considerably weak effect, where no single eQTL would be picked out. We reserve the topic of further eQTL determination in TLegene as an important direction for future study. Finally, as a methodological study, our work cannot perfectly validate these discovered eGenes for various cancers although many of the eGenes detected by TLegene could be replicated by the traditional method or PancanQTL. Therefore, our findings warranted further validations by extensive experimental studies.

## Conclusions

TLegene represents a flexible and powerful statistical method for eGene identification through transfer learning of genetic similarity shared across auxiliary and target studies.

### Supplementary Information

**Additional file 1. Table S1. ** Number of genes determined by whether the P-values of regression coefficients and TLegene-oScore are less than 0.05. **Table S2**. Number of genes determined by whether the P-values of regression coefficients and TLegene-fScore are less than 0.05.  **Table S3**. Number of genes determined by whether the P-values of regression coefficients and TLegene-aScore are less than 0.05. **Table S4**. Number of genes determined by whether the P-values of regression coefficients and TLegene-HMP are less than 0.05. **Table S5**. eGenes which identified by TLegene in the all ten TCGA cancers. **Figure S1**–**S9**. Comparison of power for the five test methods under the alternative scenarios. **Figure S10**. Upset plot represents the number of shared eGenes across the ten TCGA cancers. **Figure S11**. Bar plot represents the percentage of replicated eGenes by the traditional method using linear regression and PancanQTL, respectively. **Figure S12**. *R*^2^ distribution of SNP effects of eGenes identified in TCGA cancers. **Figure S13**. Result of the KEGG enrichment analysis of eGenes for COAD, LUAD and PAAD. **Figure S14**. Result of the GO enrichment analysis of eGenes for LUSC, LUAD, BRCA, COAD and PAAD. **Figure S15**. Enrichment of differentially expressed ones of all identified eGenes in terms of expression level across 54 GTEx tissues in BRCA and STAD. **Figure S16**. Enrichment of differentially expressed ones of all identified eGenes in terms of expression level across 54 GTEx tissues in COAD, LUAD, LUSC and PAAD. **Figure S17**. Result of the GO enrichment analysis of eGenes identified in the Geuvadis project. **Figure S18**. Enrichment of differentially expressed ones of all identified eGenes in terms of expression level across 54 GTEx tissues in Geuvadis.

## Data Availability

All data generated or analyzed during this study are included in this published article and its Additional file.

## Abbreviations

GWAS: Genome-wide association study
eQTL: Expression quantitative trait loci
SNP: Single nucleotide polymorphisms
GTEx: Genotype-tissue expression
FDR: False discover rate
PVE: Phenotypic variance explained
LD: Linkage disequilibrium
HMP: Harmonic mean *P*-value
