# Supplementary material for: Incorporating genetic similarity of auxiliary samples into eGene identification under the transfer learning framework
Source: J Transl Med. 2024 Mar 9;22:258. doi: 10.1186/s12967-024-05053-6 (PMC10924384; doi:10.1186/s12967-024-05053-6)
Supplement: Supplementary file 1 — Additional file 1. Table S1. Number of genes determined by whether the P-values of regression coefficients and TLegene-oScore are less than 0.05. Table S2. Number of genes determined by whether the P-values of regression coefficients and TLegene-fScore are less than 0.05. Table S3. Number of genes determined by whether the P-values of regression coefficients and TLegene-aScore are less than 0.05. Table S4. Number of genes determined by whether the P-values of regression coefficients and TLegene-HMP are less than 0.05. Table S5. eGenes which identified by TLegene in the all ten TCGA cancers. Figure S1–S9. Comparison of power for the five test methods under the alternative scenarios. Figure S10. Upset plot represents the number of shared eGenes across the ten TCGA cancers. Figure S11. Bar plot represents the percentage of replicated eGenes by the traditional method using linear regression and PancanQTL, respectively. Figure S12. R2 distribution of SNP effects of eGenes identified in TCGA cancers. Figure S13. Result of the KEGG enrichment analysis of eGenes for COAD, LUAD and PAAD. Figure S14. Result of the GO enrichment analysis of eGenes for LUSC, LUAD, BRCA, COAD and PAAD. Figure S15. Enrichment of differentially expressed ones of all identified eGenes in terms of expression level across 54 GTEx tissues in BRCA and STAD. Figure S16. Enrichment of differentially expressed ones of all identified eGenes in terms of expression level across 54 GTEx tissues in COAD, LUAD, LUSC and PAAD. Figure S17. Result of the GO enrichment analysis of eGenes identified in the Geuvadis project. Figure S18. Enrichment of differentially expressed ones of all identified eGenes in terms of expression level across 54 GTEx tissues in Geuvadis. [file 12967_2024_5053_MOESM1_ESM.docx]

**Additional file 1**

## Combination procedures in TLegene

### *Fisher’s combination*

The most commonly-used method for combining independent test statistics is Fisher’s combination, which simply takes the sum of -2×log(*P*) from the score tests of each individual component. It is well-known that Fisher’s method gains power when both components of *θ* and *τ* contribute to the eGene association, but loses power when only one component is significant. We refer to this test as TLegene-fScore with ‘‘f’’ standing for ‘‘Fisher”.

### *Optimally weighted linear combination*

In the optimally weighted linear combination in TLegene, we first construct *T_ρ_*=*ρ*×*U_θ_+*(1-*ρ*)×*U_τ_*, where *ρ* ∈[0, 1] controls the contribution of auxiliary samples. An intuitive way to determine the optimal weight *ρ*^*^ is to minimize the *P*-value (denoted by *P_ρ_*) based on *T_ρ_*, i.e.,$\text{ρ}^{\text{*}}\text{=}\underset{\text{ρ}\text{}\text{[0, 1]}}{\text{arg}\text{ min}}\text{\{}\text{P}_{\text{ρ}}\text{\}}$. However, no analytical form of *ρ*^*^ is available due to the complex expression of *P_ρ_*, we use numerical optimization techniques to find *ρ*^*^.

We denote the observed minimal *P*-value as $\text{P}_{\text{ρ}^{*}}^{\text{obs}}$ with *ρ*^*^ the optimal weight. To account for the fact that the minimal *P*-value is used, we calculate the *P*-value

$$\begin{matrix} \Pr(P_{\rho^{*}} \leq P_{\rho^{*}}^{obs}) = 1 - \Pr(P_{\rho} \geq P_{\rho^{*}}^{obs}, \forall\rho\in[0, 1]) \\ = 1 - \text{E}\left\{\text{I}(U_{\theta} < Q_{U_{\theta}}(1 - P_{\rho^{*}}^{obs}))\right. \\ \left.\times\Pr(U_{\tau} < \min_{\rho\neq1} \frac{1}{1 - \rho}\left[Q_{T_{\rho}}(1 - P_{\rho^{*}}^{obs}) - \rho U_{\theta}\right]\text{∣}U_{\theta})\right\} \end{matrix}$$

where *Q_U_*(*p*) is the (100*p*)^th^ quantile of the uniform random variable *U* and the expectation is evaluated with respect to *U_θ_*. For a given *ρ*, the quantile $\text{Q}_{\text{T}_{\text{p}}}\text{(1}-P_{\rho^{*}}^{\text{obs}})$ also has no analytical form; hence numerical approximation is used. In addition, the expectation can be obtained by fast numerical integration methods. We refer to this test as TLegene-oScore with ‘‘o’’ standing for ‘‘optimal’’.

### *Adaptively weighted linear combination*

The adaptively weighted linear combination is a data-adaptive generalization of Fisher’s combination. The test statistic has the form *T*=*ρ_θ_*×*Z_θ_*+*ρ_τ_*×*Z_τ_*, where $\text{Z}_{\text{θ}}\text{=}\text{-}\text{2×log(}\text{P}_{\text{θ}}\text{)}$ and $\text{Z}_{\text{τ}}\text{=-2×log(}\text{P}_{\text{τ}}\text{)}$. Under this combination, we acquire *ρ_θ_* and *ρ_τ_* determined by *Z_θ_* and *Z_τ_* via the following two formulas

$$\rho_{\theta} = \frac{Z_{\theta}}{\sqrt{Z_{\theta}^{2} + Z_{\tau}^{2}}}\text{,} \rho_{\tau} = \frac{Z_{\tau}}{\sqrt{Z_{\theta}^{2} + Z_{\tau}^{2}}}$$

Intuitively, the two weights are equivalent to the sine and cosine functions of the angle between the direction of the observed (*Z_θ_*, *Z_τ_*)∈*R*^2^ and the x-axis. The test statistic can be further simplified and expressed as $\text{T}\text{=}\text{Z}_{\text{θ}}^{\text{2}}\text{+}\text{Z}_{\text{τ}}^{\text{2}}$. This simplified form of *T* provides an insight on its asymptotic null distribution, which is the summation of squares of two independent $\text{χ}_{\text{2}}^{\text{2}}$ distributions. The numerical integration approaches can be employed in the *P*-value calculation at a low computational cost. We refer to this test as TLegene-aScore with ‘‘a’’ standing for ‘‘adaptive’’.

## Connection of some discovered eGenes with certain cancers

Previous studies provided empirical evidence for several of these identified eGenes to support their connections with certain cancers, with some examples given below. First, *ERAP2* was identified in eight cancers except for BRCA and OV. The expression and functional defects of *ERAP2* were detected in various solid tumors and hematological tumors [[1](#_ENREF_1)]. When comparing the ERAP-encoded protein between tumor and normal tissue, it was found that the gene underwent significant changes related to cancer during malignant transformation which resulted in additional losses, gains and imbalances in specific tumor tissue types and that the peptide pruning ability of tumor cells *in vivo* was thus altered [[2](#_ENREF_2)].

Second, *ANAPC13*, which was identified in OV, LIHC, PAAD, LUAD, and BRCA, was shown to be related to cancer initiation or progression [[3](#_ENREF_3)], and its down-regulation was demonstrated to be an early event in the progression of pre-invasive ductal carcinoma of the breast [[4](#_ENREF_4)]. Third, *DNAJC15*, which was detected in LIHC, PAAD, LUSC, BRCA, and LUAD, was demonstrated to inhibit Doxorubicin-resistance in breast cancer cells and could serve as a new target gene responsible for ETV7-mediated Doxorubicin-resistance [[5](#_ENREF_5)].

Fourth, *LRRC37A2*, which was identified in seven cancers except for BRCA, LIHC, and OV, might receive regulation of DNA methylation in gastric cancer cell lines; as a result, DNA hypermethylation could contribute to reduced *LRRC37A2* expression in undifferentiated and poorly differentiated gastric cancer [[6](#_ENREF_6)].

## Characteristics of eGenes and functional enrichment analysis

We performed GO and KEGG enrichment analysis for these eGenes using the clusterProfiler package [[7](#_ENREF_7)], and identified several enriched pathways for BRCA, LUAD, LUSC, PAAD and STAD (Figures S11-12). The biological processes (BP) of these genes were significantly enriched in the processing and presentation of peptide antigens, the assembly of MHC class II protein complexes, the assembly of protein complexes, and the metabolism of purine nucleosides. For the cell component (CC) term, these genes were enriched in MHC protein complexes, endoplasmic reticulum membranes and mitochondrial matrices. For the molecular function (MF) term, these genes were mainly enriched in oxidoreductase activity, acting on donor aldehyde or oxidative groups and amide binding.

The KEGG enrichment analysis shows that these genes are enriched in antigen processing and presentation, hematopoietic cell lineage, allograft rejection, and cytochrome P450 (CYC P450). The CYP family was shown to be involved in the metabolism of many procarcinogens, producing secondary metabolites that could cause DNA damage, produce chemical adducts and act to activate or inactivate anticancer drugs.

For BRCA, recognition of new shared breast cancer antigens by T cells was frequently observed in the peripheral blood of breast cancer patients [[8](#_ENREF_8)], and breast bead protein remained a useful marker for detecting breast cancer cells in peripheral blood [[9](#_ENREF_9)]. For STAD, some studies confirmed the bidirectional activity of the central nervous system (CNS) and the gastrointestinal tract, which played an important role in intestinal motility, absorption, endocrine and immune functions, and in maintaining the stability of the internal environment of the digestive tract. Within the gastrointestinal stem cell ecotone, nerve growth factor can lead to gastric carcinogenesis through abnormal cholinergic signaling [[10](#_ENREF_10)].

In addition, we performed the GO and KEGG enrichment analysis for eGenes identified in Geuvadis, and revealed several enriched pathways (Figure S15). For the BP term, these eGenes were enriched in cellular amide metabolic processes, biosynthetic processes of organic nitrogen compounds, redox processes and intracellular transport. For the CC term, these eGenes were enriched in mitochondria, ribosomal subunits, cell membrane and ribosome. For the MF term, these eGenes were enriched in sphingolipid phosphodiesterase activity and oxide reductase activity.

## Table S1. Number of genes determined by whether the P-values of regression coefficients and TLegene-oScore are less than 0.05

| cancer | FDR<=0.05  oScore<=0.05 | FDR<=0.05  oScore>0.05 | FDR>0.05  oScore<=0.05 | FDR>0.05  oScore>0.05 | *P* |
| --- | --- | --- | --- | --- | --- |
| ACC | 310 | 4,919 | 40 | 1,628 | 1.06×10^-8^ |
| BRCA | 631 | 2,560 | 138 | 907 | 2.00×10^-6^ |
| COAD | 602 | 4,122 | 66 | 1,431 | 3.63×10^-25^ |
| LIHC | 396 | 3,622 | 56 | 1,244 | 2.32×10^-2^ |
| LUAD | 722 | 3,979 | 105 | 1,260 | 3.70×10^-13^ |
| LUSC | 521 | 3,628 | 86 | 1,229 | 3.12×10^-10^ |
| OV | 394 | 2,782 | 109 | 1,125 | 8.07×10^-4^ |
| PAAD | 575 | 4,492 | 75 | 1,567 | 6.91×10^-16^ |
| STAD | 496 | 3,680 | 79 | 1,227 | 1.98×10^-9^ |
| UCEC | 142 | 1,993 | 21 | 705 | 1.61×10^-4^ |

## Table S2. Number of genes determined by whether the P-values of regression coefficients and TLegene-fScore are less than 0.05

| cancer | FDR<=0.05  fScore<=0.05 | FDR<=0.05  fScore>0.05 | FDR>0.05  fScore<=0.05 | FDR>0.05  fScore>0.05 | *P* |
| --- | --- | --- | --- | --- | --- |
| ACC | 286 | 4,943 | 35 | 1,633 | 1.26×10^-8^ |
| BRCA | 683 | 2,508 | 141 | 904 | 2.05×10^-8^ |
| COAD | 646 | 4,078 | 71 | 1,426 | 4.07×10^-21^ |
| LIHC | 396 | 3,622 | 63 | 1,237 | 2.26×10^-8^ |
| LUAD | 771 | 3,930 | 108 | 1,257 | 4.39×10^-15^ |
| LUSC | 548 | 3,601 | 90 | 1,225 | 3.80×10^-10^ |
| OV | 389 | 2,787 | 113 | 1,121 | 3.72×10^-3^ |
| PAAD | 593 | 4,474 | 74 | 1,568 | 2.46×10^-17^ |
| STAD | 518 | 3,658 | 78 | 1,228 | 7.17×10^-11^ |
| UCEC | 149 | 1,986 | 23 | 703 | 1.9×10^-4^ |

## Table S3. Number of genes determined by whether the P-values of regression coefficients and TLegene-aScore are less than 0.05

| cancer | FDR<=0.05  aScore<=0.05 | FDR<=0.05  aScore>0.05 | FDR>0.05  aScore<=0.05 | FDR>0.05  aScore>0.05 | *P* |
| --- | --- | --- | --- | --- | --- |
| ACC | 284 | 8,249 | 21 | 2,609 | 1.26×10^-8^ |
| BRCA | 262 | 5,003 | 44 | 2,474 | 2.05×10^-8^ |
| COAD | 325 | 8,179 | 54 | 2,977 | 4.07×10^-21^ |
| LIHC | 248 | 6,148 | 37 | 2,139 | 5.50×10^-11^ |
| LUAD | 332 | 7,585 | 45 | 2,654 | 8.30×10^-13^ |
| LUSC | 264 | 6,740 | 41 | 2,392 | 8.01×10^-10^ |
| OV | 380 | 4,949 | 141 | 2,249 | 3.69×10^-4^ |
| PAAD | 240 | 7,665 | 28 | 2,793 | 1.99×10^-17^ |
| STAD | 302 | 6,194 | 84 | 2,666 | 4.18×10^-10^ |
| UCEC | 392 | 7,694 | 61 | 2,799 | 5.00×10^-6^ |

## Table S4. Number of genes determined by whether the P-values of regression coefficients and TLegene-HMP are less than 0.05

| cancer | FDR<=0.05  aScore<=0.05 | FDR<=0.05  aScore>0.05 | FDR>0.05  aScore<=0.05 | FDR>0.05  aScore>0.05 | *P* |
| --- | --- | --- | --- | --- | --- |
| ACC | 252 | 4,977 | 26 | 16,42 | 1.45×10^-12^ |
| BRCA | 614 | 2,577 | 134 | 911 | 3.01×10^-12^ |
| COAD | 569 | 4,155 | 55 | 1,442 | 2.21×10^-11^ |
| LIHC | 352 | 3,666 | 48 | 1,252 | 5.35×10^-8^ |
| LUAD | 676 | 4,025 | 100 | 1,265 | 3.66×10^-12^ |
| LUSC | 486 | 3,663 | 76 | 1,239 | 4.64×10^-8^ |
| OV | 357 | 2,819 | 100 | 1134 | 2.63×10^-2^ |
| PAAD | 532 | 4,535 | 62 | 1,580 | 2.16×10^-10^ |
| STAD | 449 | 3,727 | 68 | 1,238 | 5.39×10^-7^ |
| UCEC | 251 | 4,397 | 43 | 1,608 | 1.38×10^-12^ |

## Table S5. eGenes which identified by TLegene in the all ten TCGA cancers

| type | gene |
| --- | --- |
| *G*_1_ | *A4GNT ABCC13 ACCS ACTN3 ACTR8 ADAL ADH4 AGA ALDH5A1 ALMS1P ALS2CR12 ANKRD36B AS3MT ATP6V1E2 BCO2 C14orf149 C16orf88 C2orf54 C2orf84 C3orf70 CAT CCDC111 CCDC82 CDA CDK2AP1 CRIPT CST2 DIRC1 DISP2 EXOSC6 FOLR3 FUT2 G6PC2 GATC GBP7 GJB5 GNMT GNRHR GPX7 HIBCH HLA-DPB2 HYAL3 IFI27L1 IFITM4P KDELC2 LCN8 LNP1 MANBA MCPH1 MRPL39 MYLK2 NDUFAF1 NDUFS5 NOM1 NOMO3 OPN4 ORMDL1 PCDHA10 PDHB PFN4 PHOSPHO2 PHYHD1 PM20D1 PTPN5 RBM44 RFT1 RPS26 RRP7A RTN4 SLC25A1 SLC38A11 SMTNL1 SNX31 SPINK6 SURF1 TAS2R20 TCTN2 TDGF1 TDH TFB1M TMED6 TPSD1 TPTE2P3 TREX1 WDSUB1 ZFAND2A ZNF323 ZNF718* |
| *G*_2_ | *ACSM1 ALDH8A1 AMIGO1 ARHGEF35 ARL16 C10orf107 C15orf57 C17orf97 C2orf63 C7orf29 CDC26 DHFR ESPNL FOXRED1 HCG22 HCG4 HEATR4 HLA-DRB1 HLA-DRB5HLA-DRB6 HLA-L ITGB3BP LILRA3 LIPT1 LRRC61 MGMT NUDT13 PEX6 PKDREJ PSORS1C1 SERPIND1 SLC5A11 SOHLH2 SYPL2 TRIM63 TRMT61B WFDC3 ZNF215 ZNF79 C22orf34 C9orf43 CCBL2 CCDC163P EFCAB2 EFHB GSTM3 HLA-DQA2 IQCB1 KRT1 NSA2 PAX8 STAG3L4 STOX1 TIMM10 TRAPPC4 TTC32 WBSCR27 WFIKKN1 ZFP57 CTSW FAM118A GLIPR1L2 MS4A14 PSORS1C3 RPL23AP7 TSPAN10 ANAPC13 C21orf56 DNAJC15 GBP3 LDHC PYROXD2 FAHD1 GSTT2 HSD17B12 ULK4 WARS2 DDX11L2 LRRC37A2 PPIL3 ERAP2* |

NOTE：*G*_1_ represents the eGene identified only in one TCGA cancer, *G*_2_ represents the eGene identified in at least two cancers


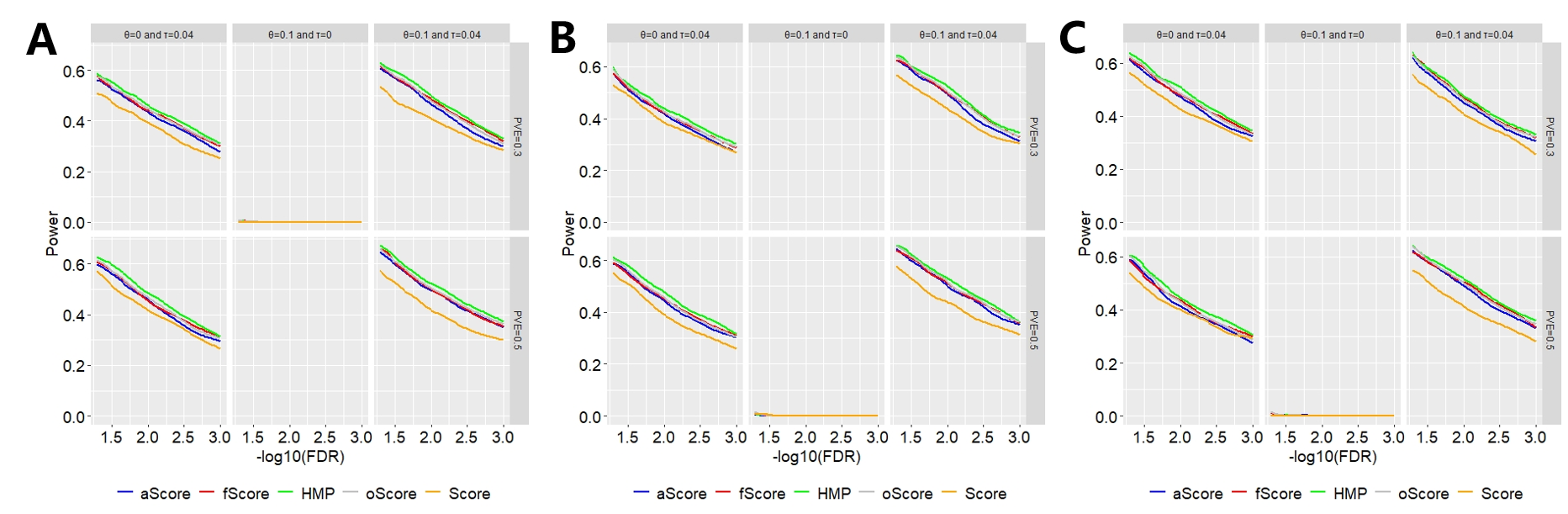


## Figure S1. Comparison of power for the five test methods under the alternative scenarios. Here, the PVE in the auxiliary study was set to 0.3 (top) or 0.5 (bottom), the sample size of the target study was 300 and the sample size of the auxiliary study was 165, *θ*=0.1 or/and τ=0.04. (A) 30% of SNPs were null; (B) 50% of SNPs were null; (C) 70% of SNPs were null.


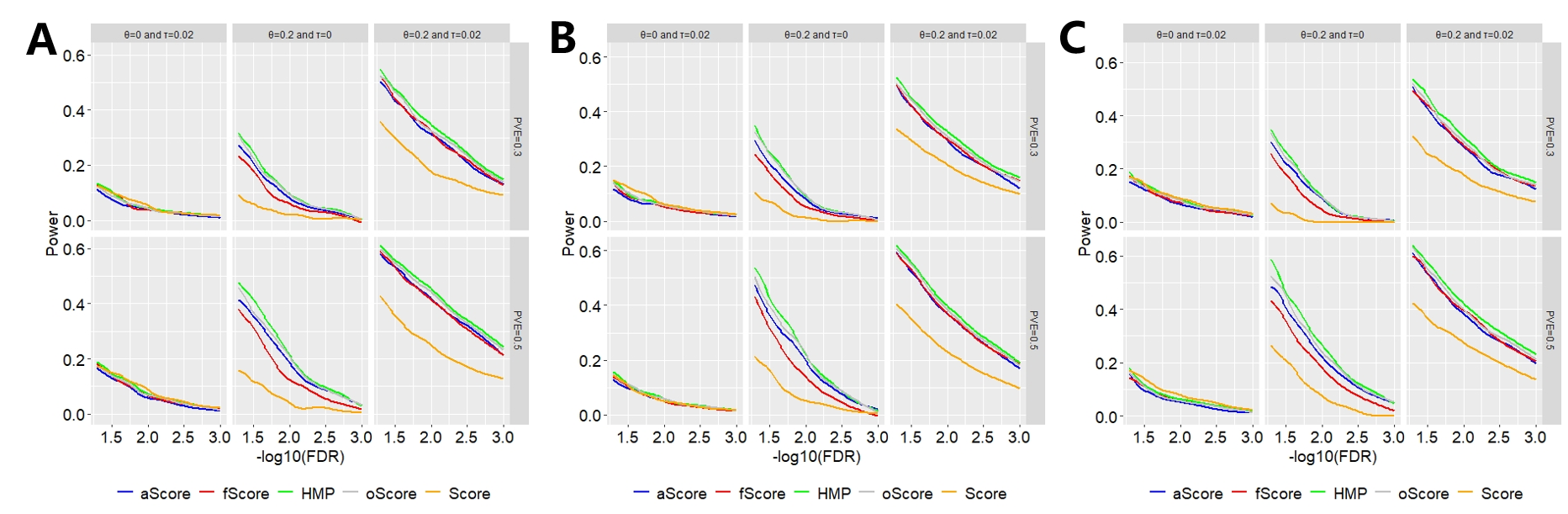


## Figure S2. Comparison of power for the five test methods under the alternative scenarios. Here, the PVE in the auxiliary study was set to 0.3 (top) or 0.5 (bottom), the sample size of the target study was 300 and the sample size of the auxiliary study was 165, *θ*=0.2 or/and τ=0.02. (A) 30% of SNPs were null; (B) 50% of SNPs were null; (C) 70% of SNPs were null.


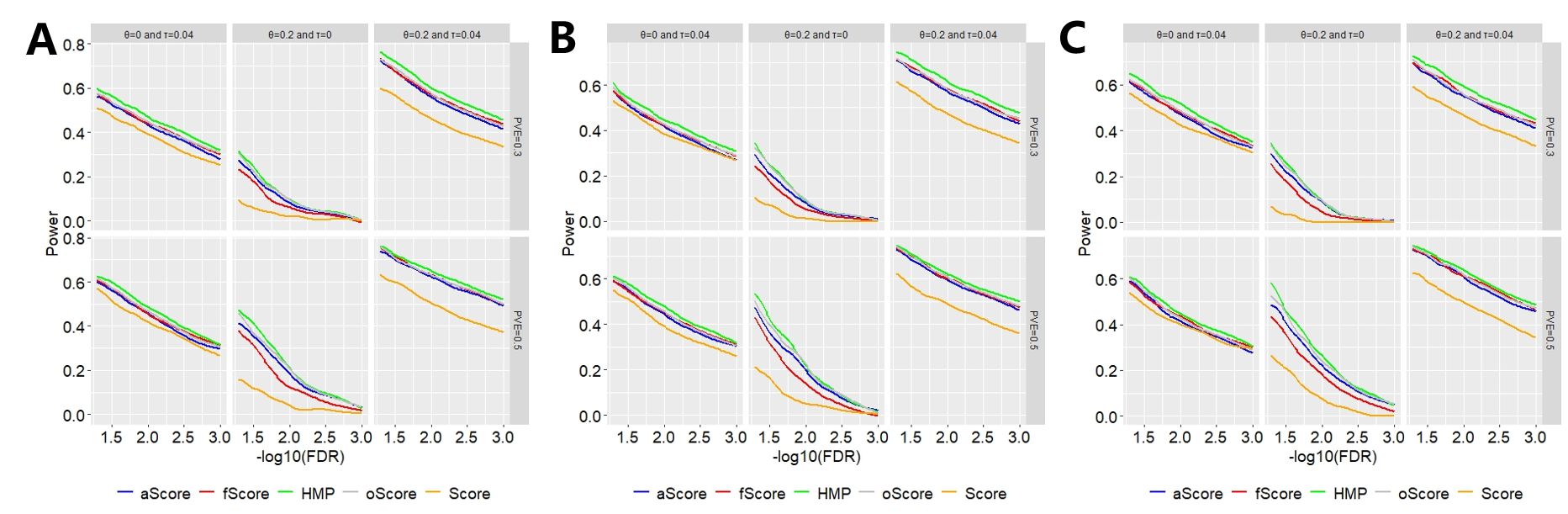


Figure S3. Comparison of power for the five test methods under the alternative scenarios. Here, the PVE in the auxiliary study was set to 0.3 (top) or 0.5 (bottom), the sample size of the target study was 300 and the sample size of the auxiliary study was 165, *θ*=0.2 or/and τ=0.04. (A) 30% of SNPs were null; (B) 50% of SNPs were null; (C) 70% of SNPs were null.


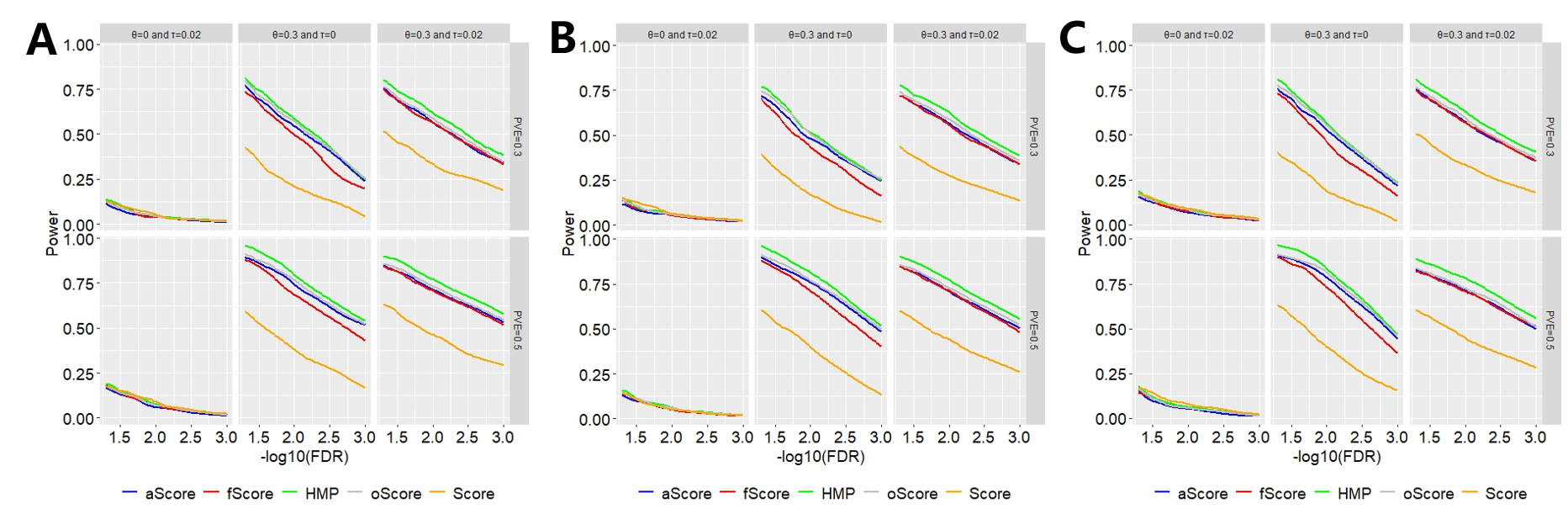


Figure S4. Comparison of power for the five test methods under the alternative scenarios. Here, the PVE in the auxiliary study was set to 0.3 (top) or 0.5 (bottom), the sample size of the target study was 300 and the sample size of the auxiliary study was 165, *θ*=0.3 or/and τ=0.02. (A) 30% of SNPs were null; (B) 50% of SNPs were null; (C) 70% of SNPs were null.


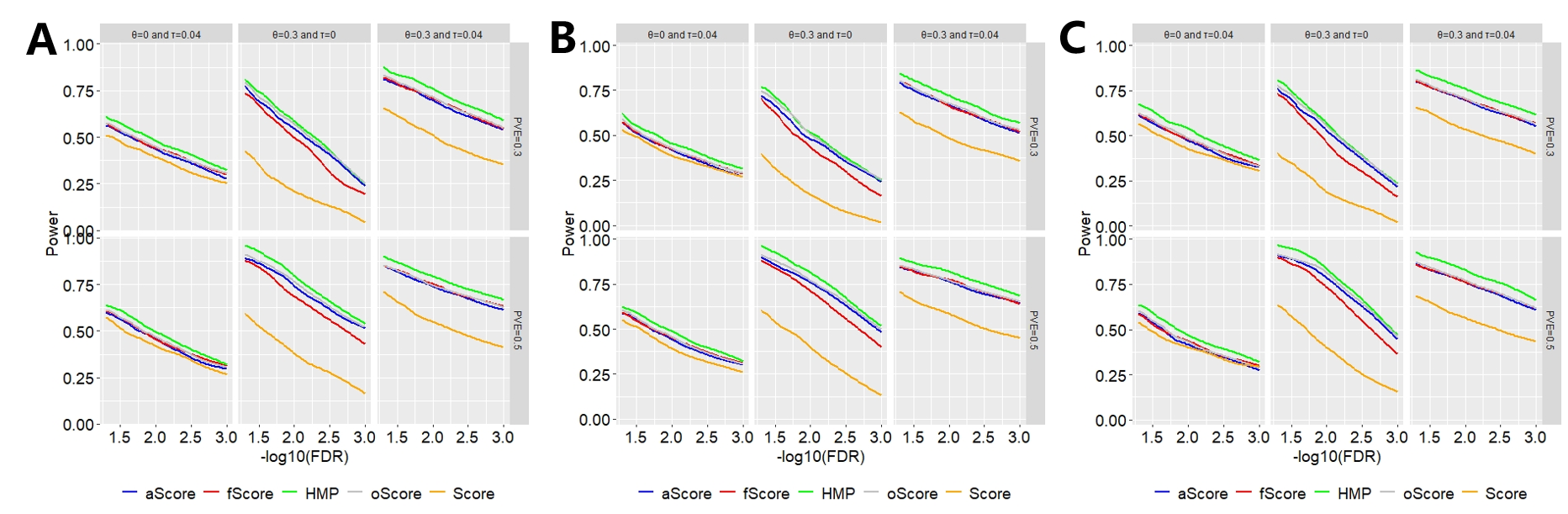


Figure S5. Comparison of power for the five test methods under the alternative scenarios. Here, the PVE in the auxiliary study was set to 0.3 (top) or 0.5 (bottom), the sample size of the target study was 300 and the sample size of the auxiliary study was 165, *θ*=0.3 or/and τ=0.04. (A) 30% of SNPs were null; (B) 50% of SNPs were null; (C) 70% of SNPs were null.


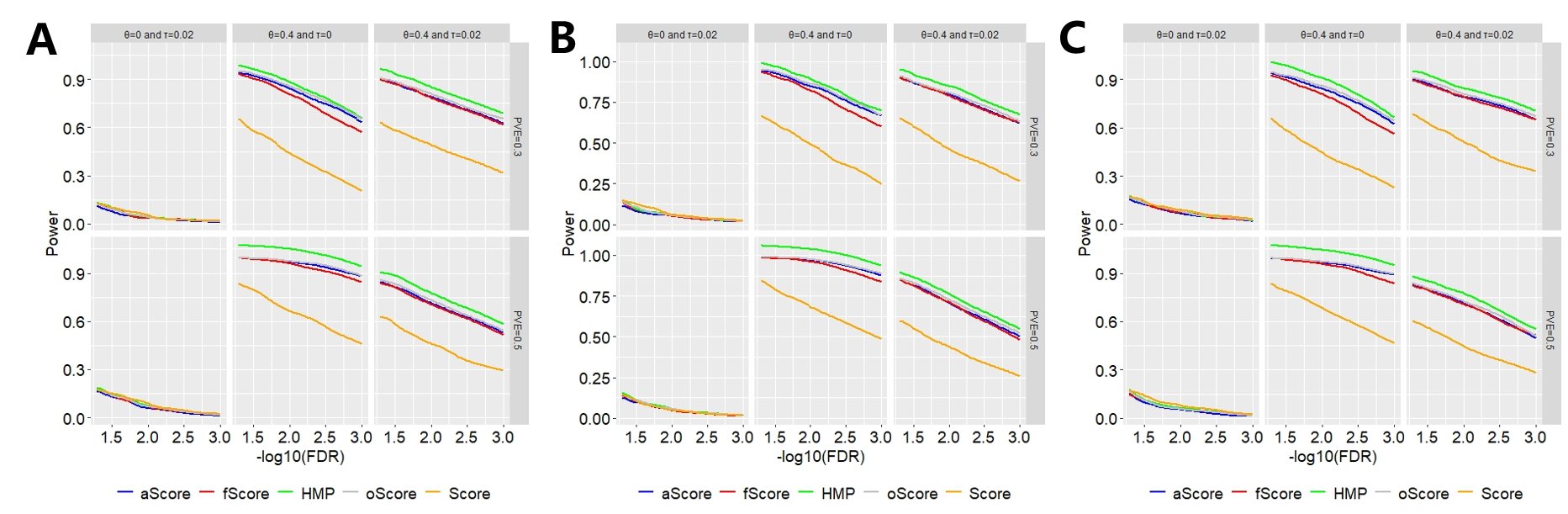


Figure S6. Comparison of power for the five test methods under the alternative scenarios. Here, the PVE in the auxiliary study was set to 0.3 (top) or 0.5 (bottom), the sample size of the target study was 300 and the sample size of the auxiliary study was 165, *θ*=0.4 or/and τ=0.02. (A) 30% of SNPs were null; (B) 50% of SNPs were null; (C) 70% of SNPs were null.


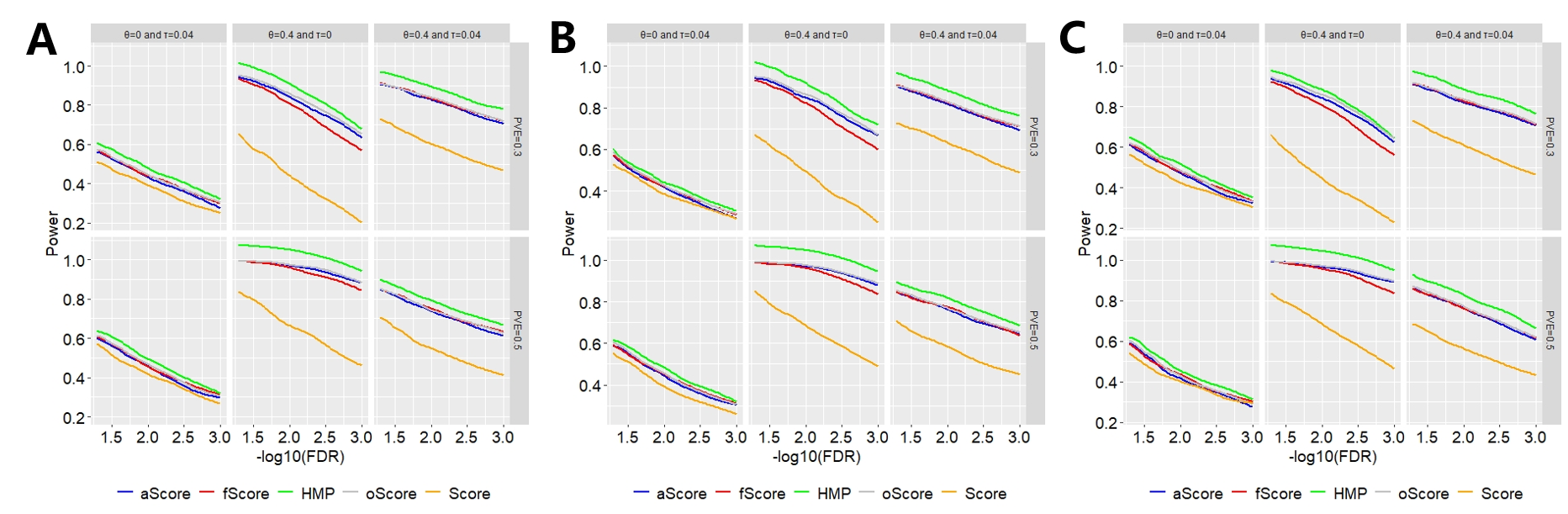


Figure S7. Comparison of power for the five test methods under the alternative scenarios. Here, the PVE in the auxiliary study was set to 0.3 (top) or 0.5 (bottom), the sample size of the target study was 300 and the sample size of the auxiliary study was 165, *θ*=0.3 or/and τ=0.04. (A) 30% of SNPs were null; (B) 50% of SNPs were null; (C) 70% of SNPs were null.


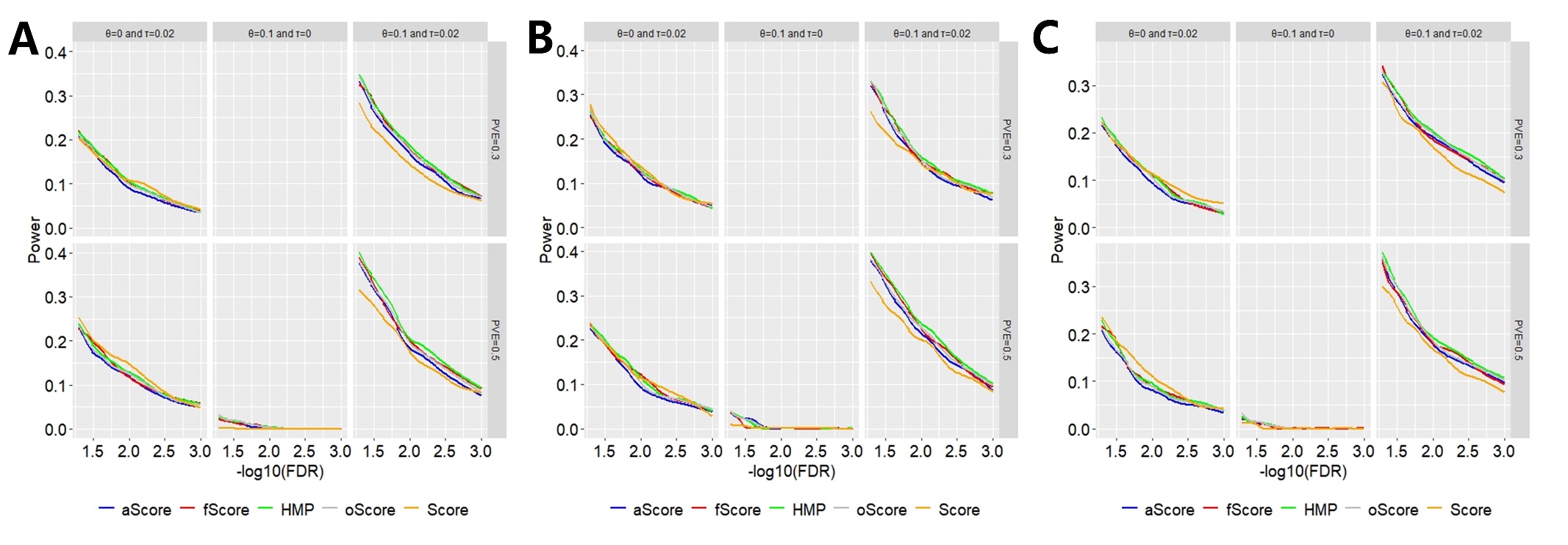


## Figure S8. Comparison of power for the five test methods under the alternative scenarios. Here, the PVE in the auxiliary study was set to 0.3 (top) or 0.5 (bottom), the sample size of the target study was 100 and the sample size of the auxiliary study was 400, *θ*=0.1 or/and τ=0.02. (A) 30% of SNPs were null; (B) 50% of SNPs were null; (C) 70% of SNPs were null.


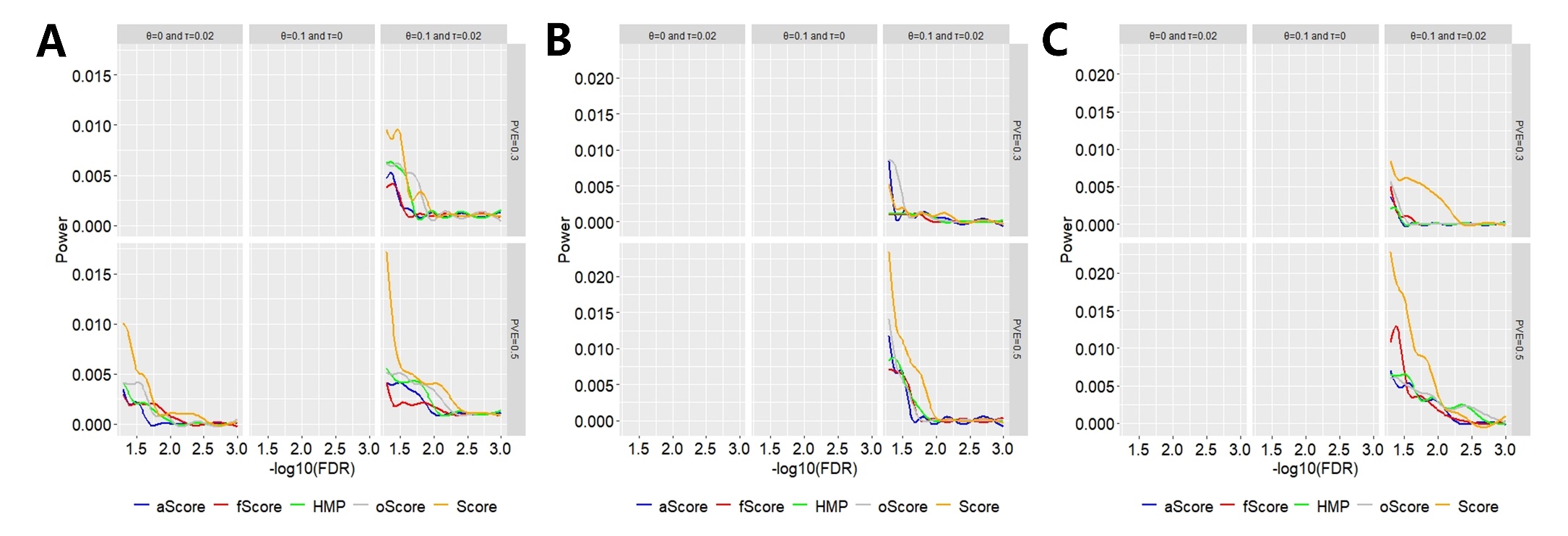


## Figure S9. Comparison of power for the five test methods under the alternative scenarios. Here, the PVE in the auxiliary study was set to 0.3 (top) or 0.5 (bottom), the sample size of the target study was 400 and the sample size of the auxiliary study was 100, θ=0.1 or/and τ=0.02. (A) 30% of SNPs were null; (B) 50% of SNPs were null; (C) 70% of SNPs were null.


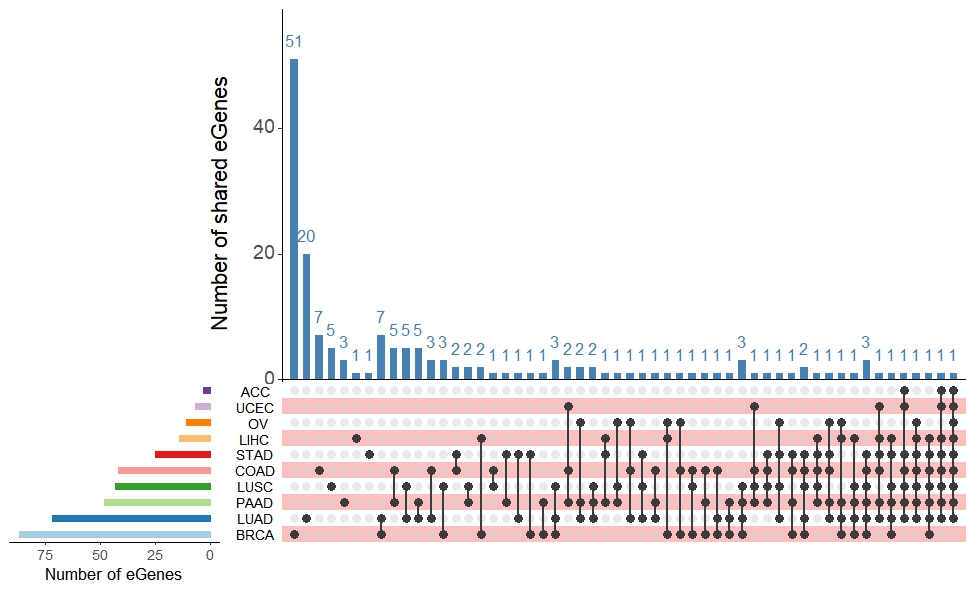


## Figure S10. Upset plot represents the number of shared eGenes across the ten TCGA cancers.


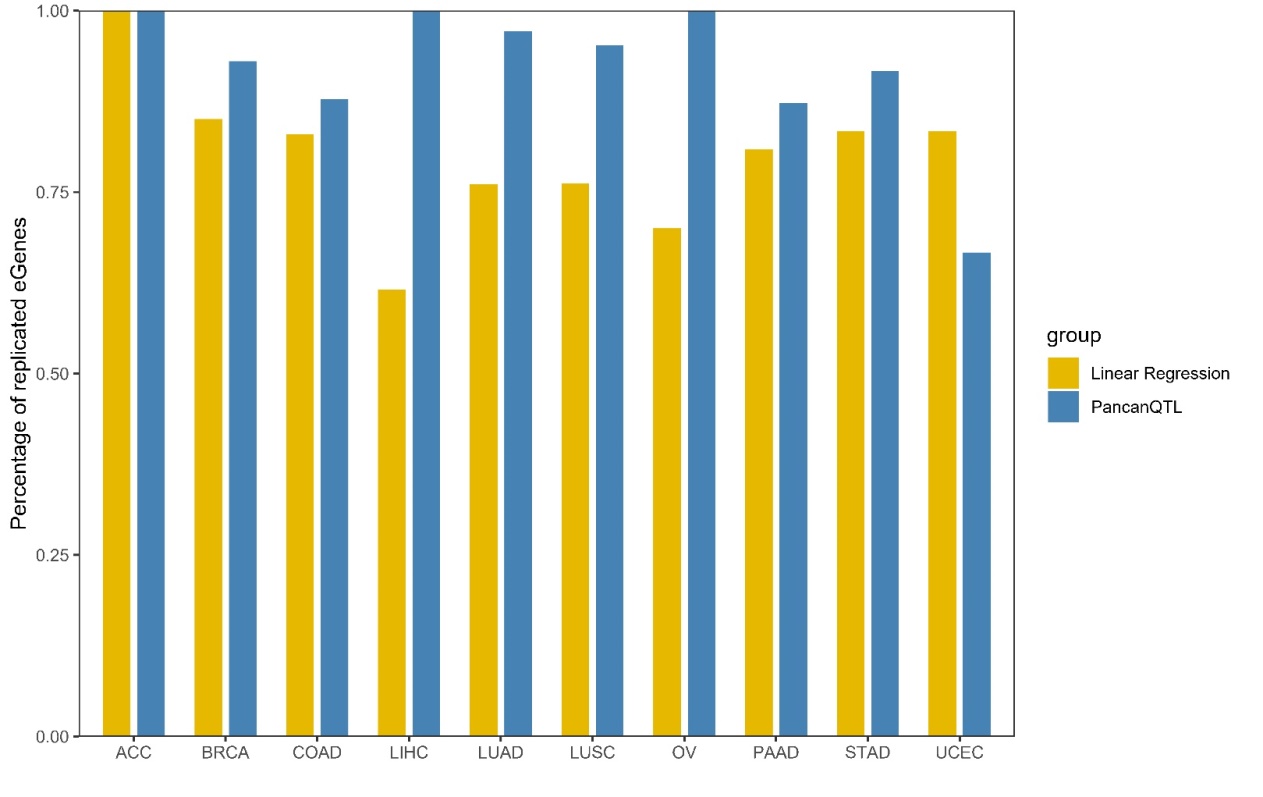


## Figure S11. Bar plot represents the percentage of replicated eGenes by the traditional method using linear regression and PancanQTL, respectively.


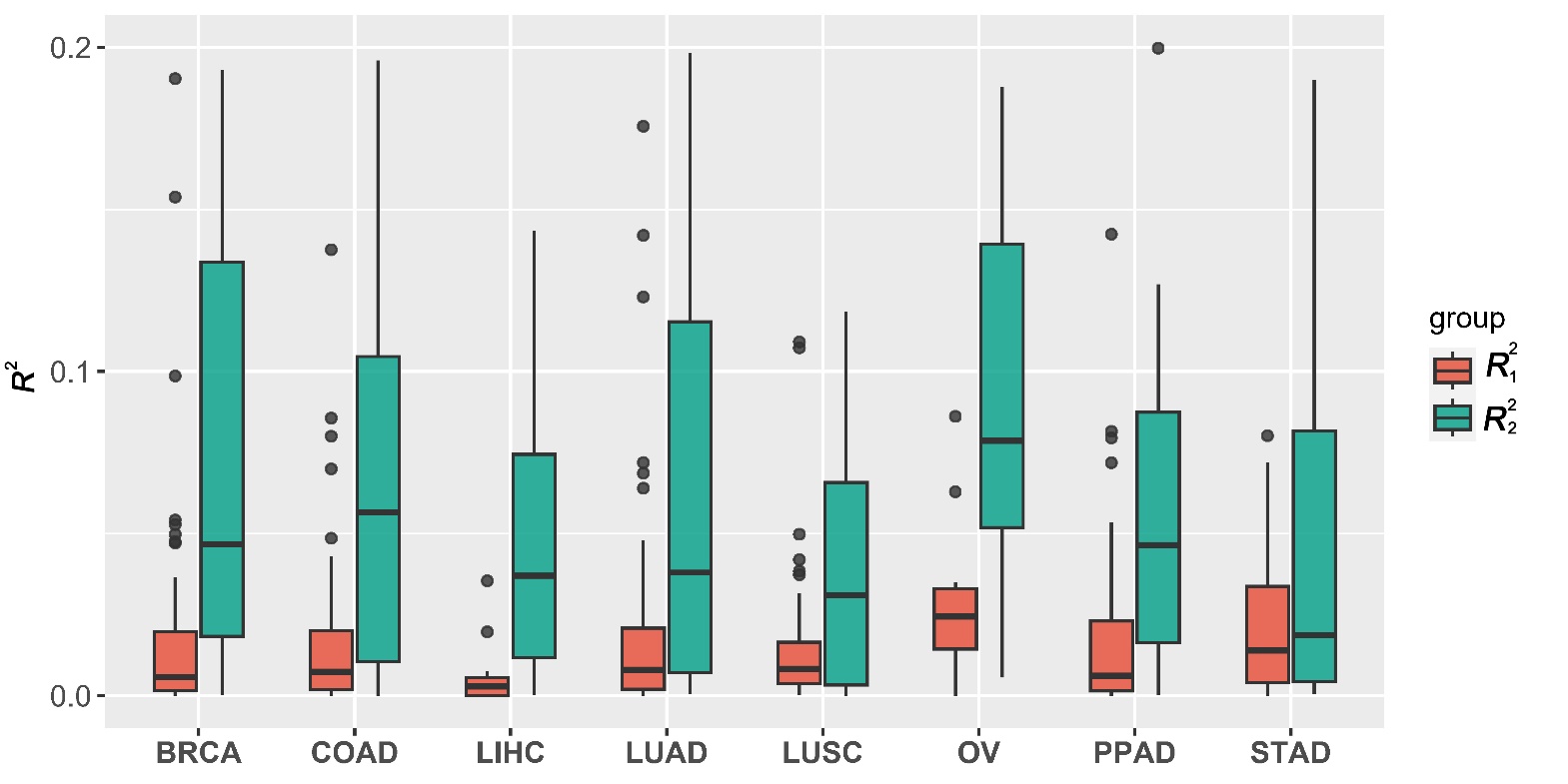


## Figure S12. *R*^2^ distribution of SNP effects of eGenes identified in TCGA cancers; *R*^2^: the determination coefficient of the cis-SNPs effects for each gene; $\text{R}_{\text{1}}^{\text{2}}$: estimated using insignificant cis-SNPs of each eGene; $\text{R}_{\text{2}}^{\text{2}}$: estimated using significant cis-SNPs of each eGene. ACC is not shown here because no eGenes were identified.


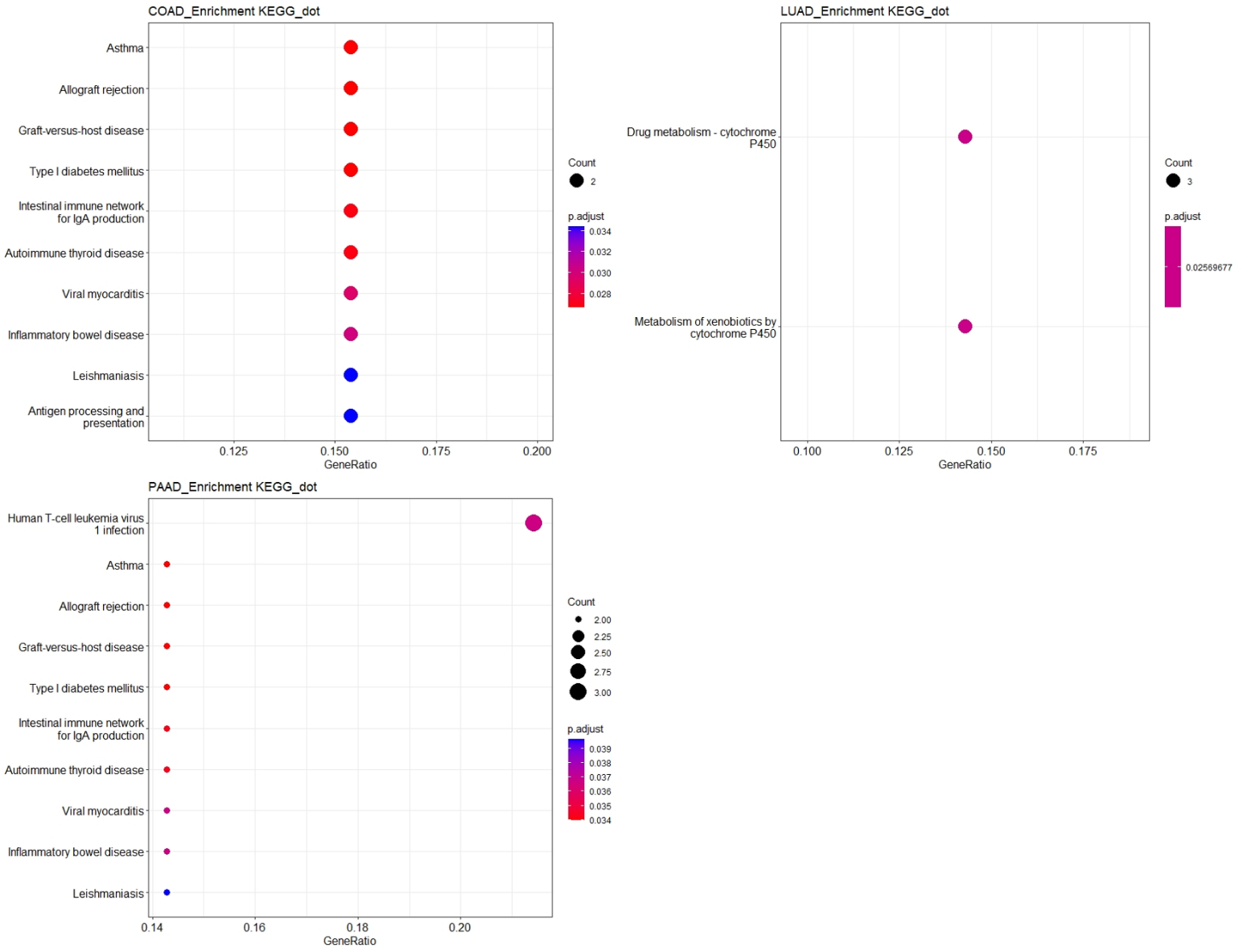


## Figure S13. Result of the KEGG enrichment analysis of eGenes for COAD, LUAD and PAAD.


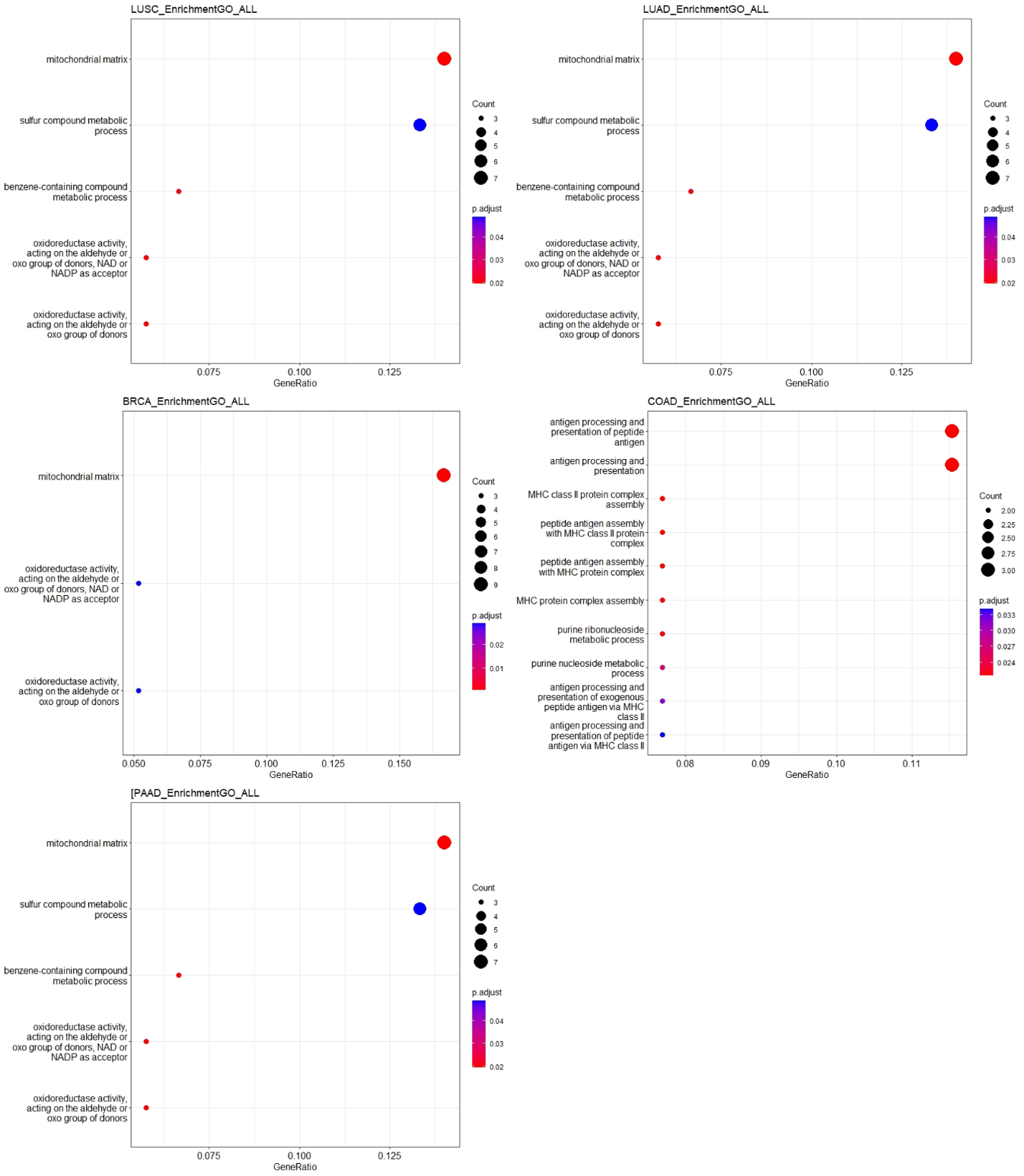
.
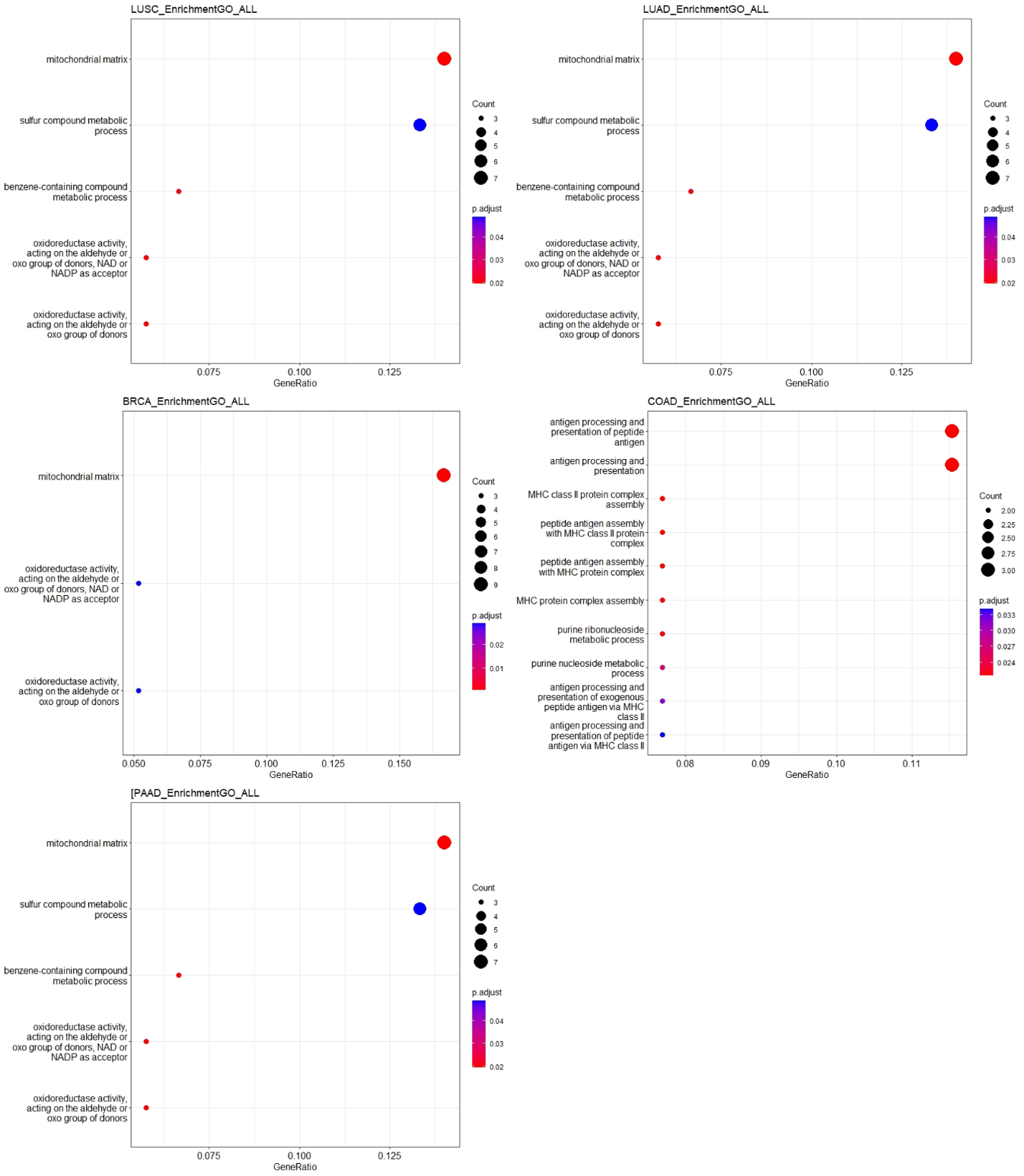


## Figure S14. Result of the GO enrichment analysis of eGenes for LUSC, LUAD, BRCA, COAD and PAAD.


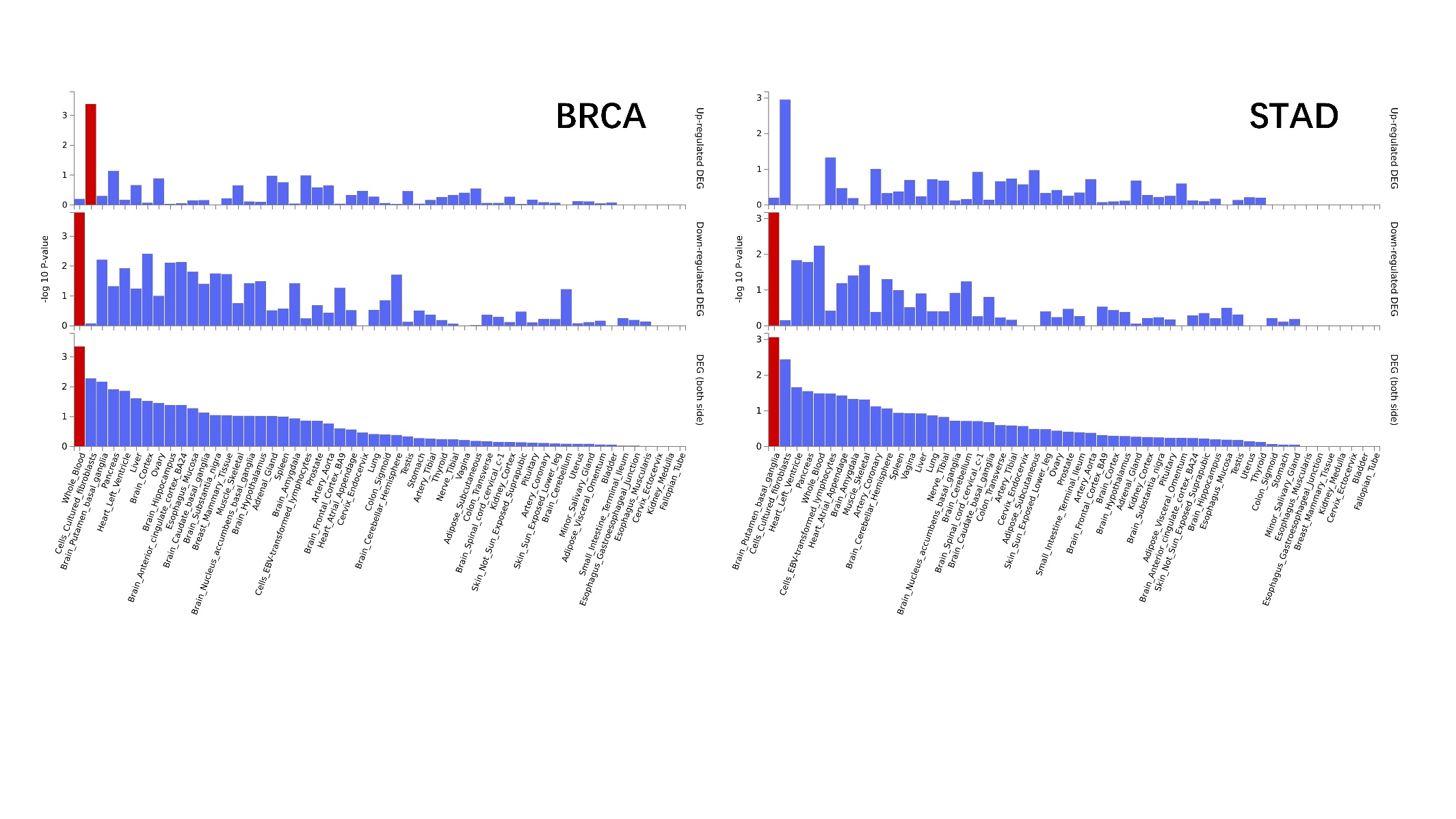


## Figure S15. Enrichment of differentially expressed ones of all identified eGenes in terms of expression level across 54 GTEx tissues in BRCA and STAD. *P* values are shown in the y-axis with a scale of -log10, the bars in red represent significant enrichment with Bonferroni adjustment for multiple hypothesis testing.


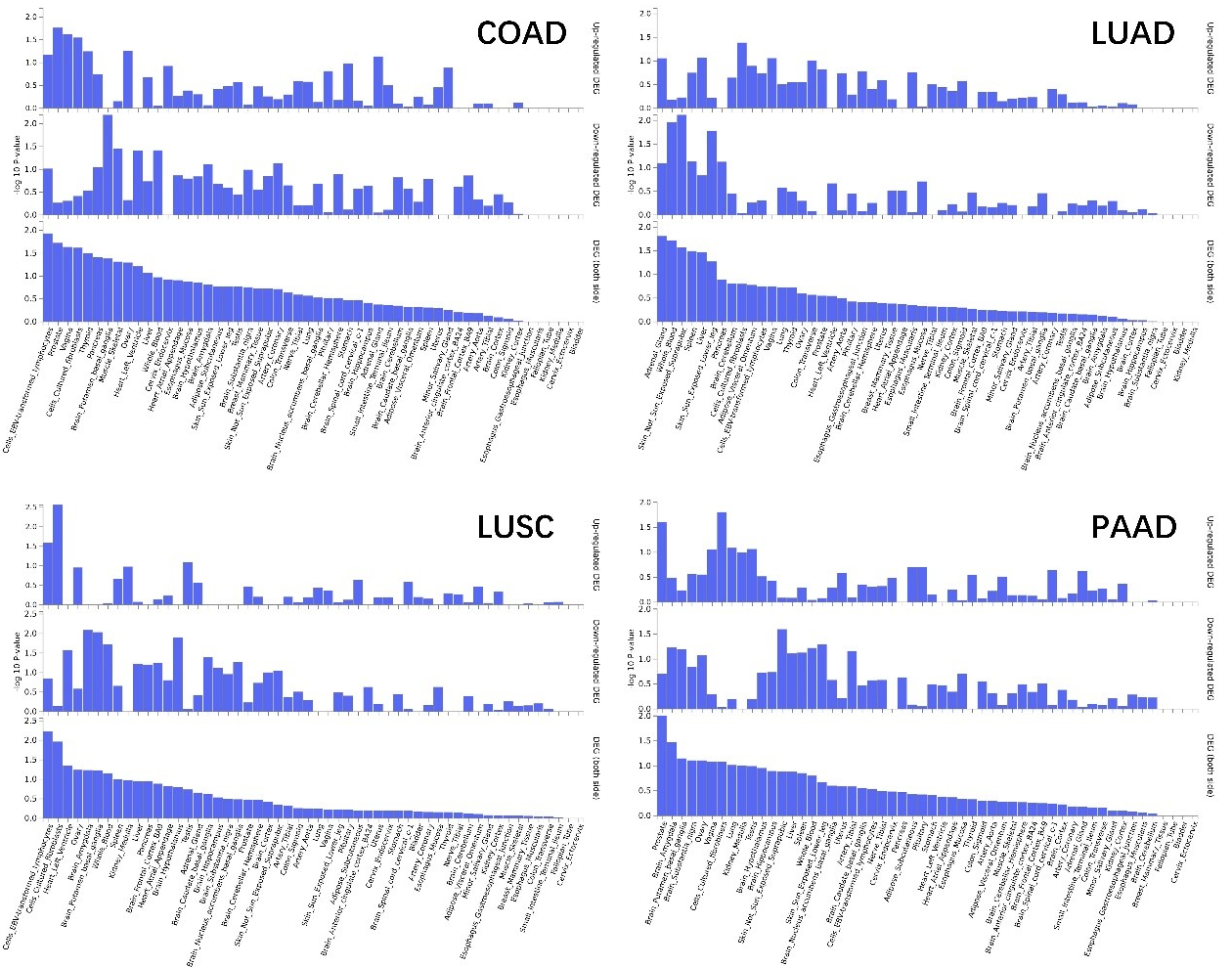


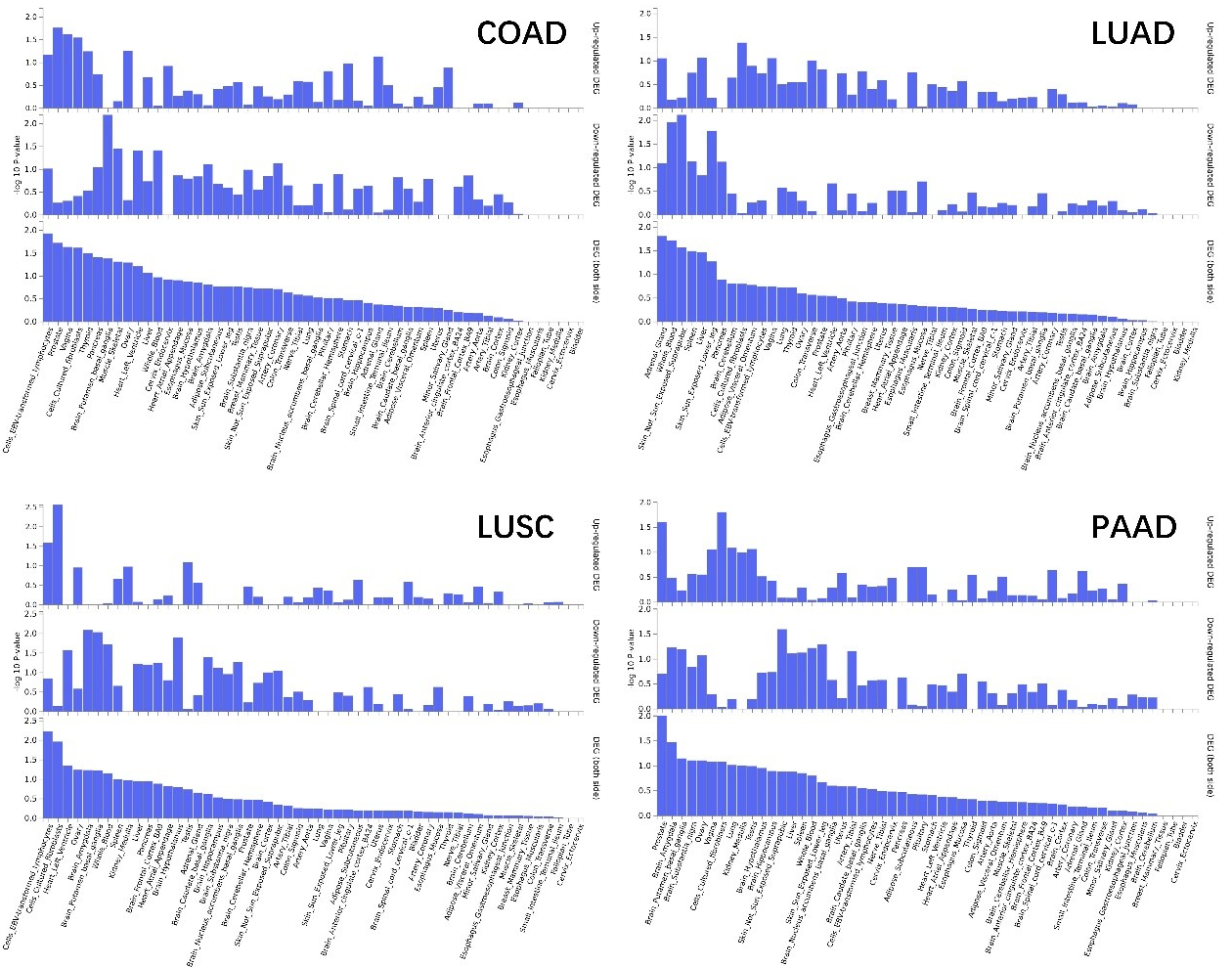


## Figure S16. Enrichment of differentially expressed ones of all identified eGenes in terms of expression level across 54 GTEx tissues in COAD, LUAD, LUSC and PAAD. *P*-values are shown in the y-axis with a scale of -log10, the bars in red represent significant enrichment with Bonferroni adjustment for multiple hypothesis testing.


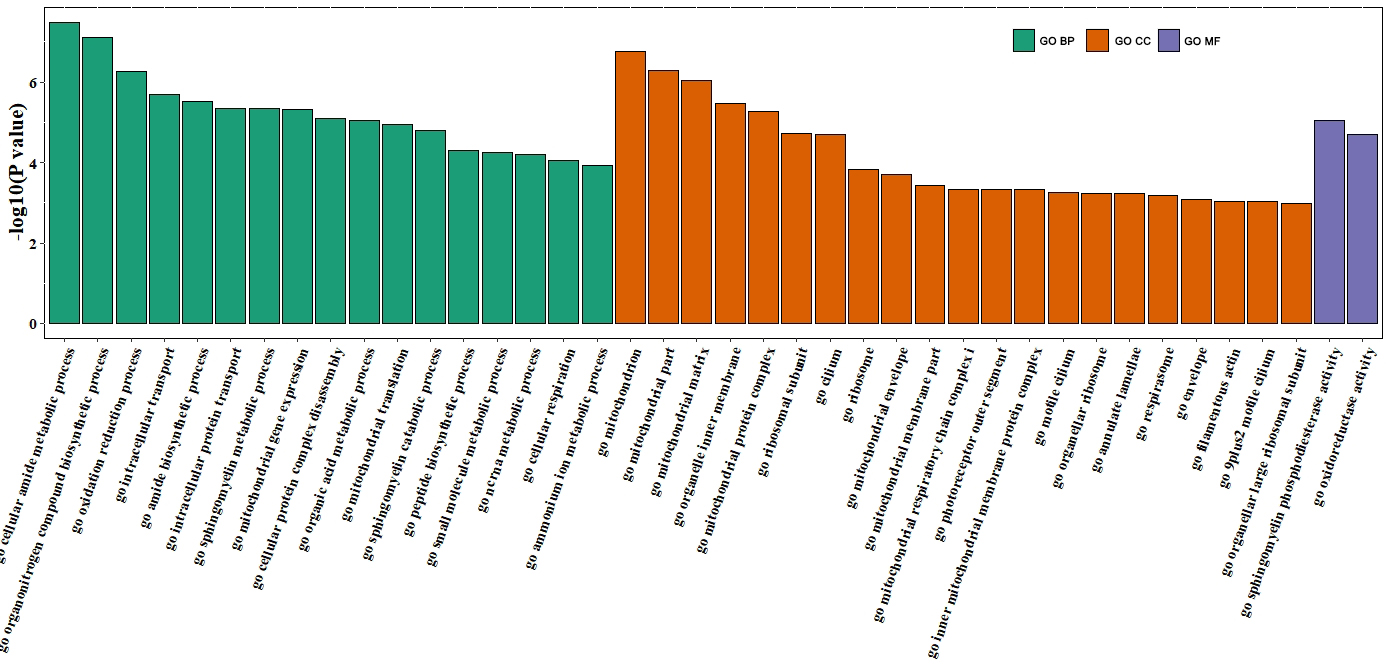


## Figure S17. Result of the GO enrichment analysis of eGenes identified in the Geuvadis project.


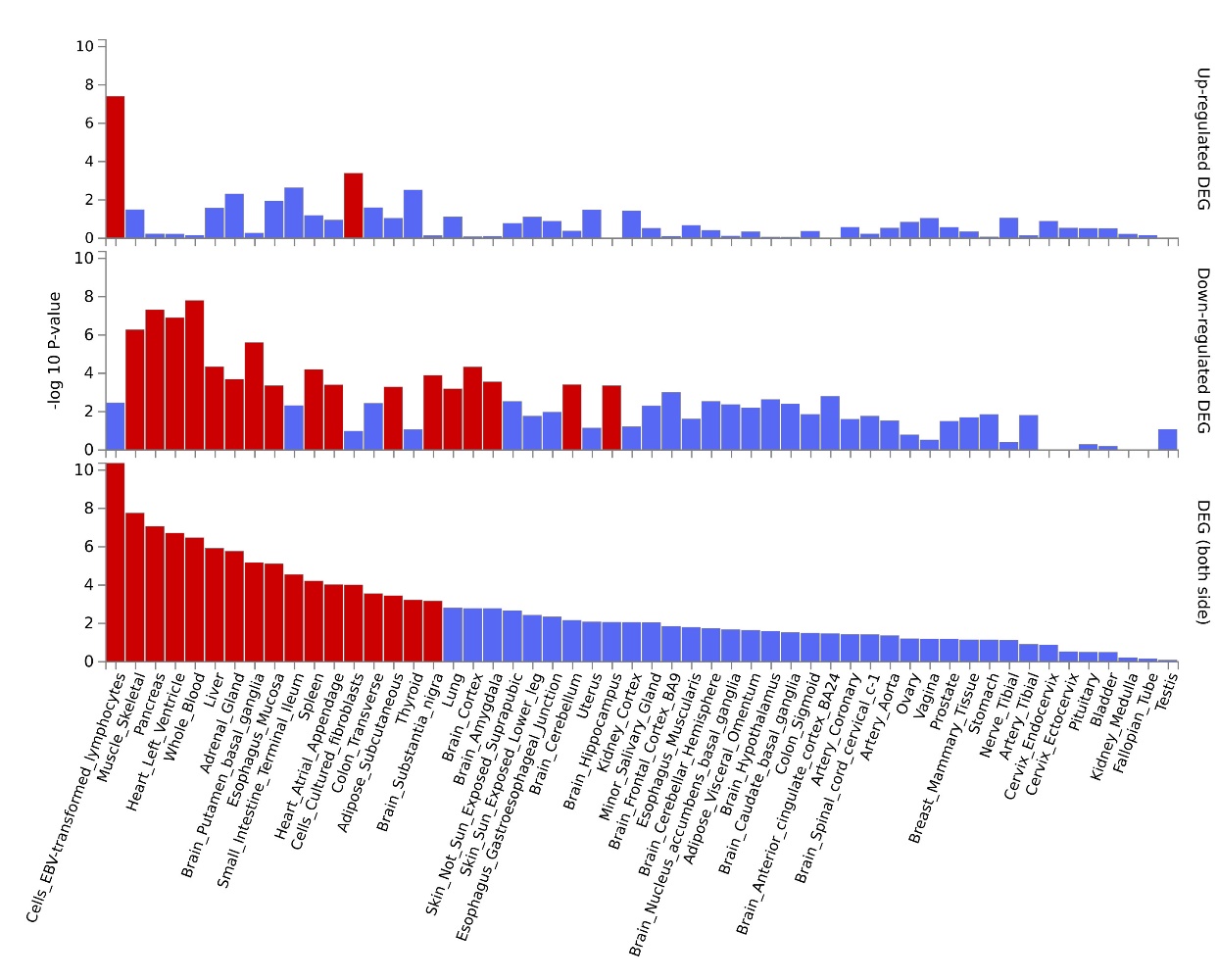


## Figure S18. Enrichment of differentially expressed ones of all identified eGenes in terms of expression level across 54 GTEx tissues in Geuvadis. *P*-values are shown in the y-axis with a scale of -log10, the bars in red represent significant enrichment with Bonferroni adjustment for multiple hypothesis testing.

## References

1. Stratikos E, Stamogiannos A, Zervoudi E, Fruci D: **A role for naturally occurring alleles of endoplasmic reticulum aminopeptidases in tumor immunity and cancer pre-disposition.** *Front Oncol.* 2014, **4:**363.

2. Fruci D, Giacomini P, Nicotra MR, Forloni M, Fraioli R, Saveanu L, van Endert P, Natali PG: **Altered expression of endoplasmic reticulum aminopeptidases ERAP1 and ERAP2 in transformed non-lymphoid human tissues.** *J Cell Physiol.* 2008, **216:**742-749.

3. Valdiglesias V, Fernández-Tajes J, Pásaro E, Méndez J, Laffon B: **Identification of differentially expressed genes in SHSY5Y cells exposed to okadaic acid by suppression subtractive hybridization.** *BMC Genomics.* 2012, **13:**46.

4. Sens-Abuázar C, Napolitano EFE, Osório CA, Krepischi AC, Ricca TI, Castro NP, da Cunha IW, Maciel Mdo S, Rosenberg C, Brentani MM, et al: **Down-regulation of ANAPC13 and CLTCL1: Early Events in the Progression of Preinvasive Ductal Carcinoma of the Breast.** *Transl Oncol.* 2012, **5:**113-123.

5. Alessandrini F, Pezzè L, Menendez D, Resnick MA, Ciribilli Y: **ETV7-Mediated DNAJC15 Repression Leads to Doxorubicin Resistance in Breast Cancer Cells.** *Neoplasia.* 2018, **20:**857-870.

6. Wisnieski F, Geraldis JC, Santos LC, Leal MF, Calcagno DQ, Gigek CO, Chen ES, Anauate AC, Artigiani R, Demachki S, et al: **Differential regulation of LRRC37A2 in gastric cancer by DNA methylation.** *Epigenetics.* 2022, **17:**110-116.

7. Yu G, Wang LG, Han Y, He QY: **clusterProfiler: an R package for comparing biological themes among gene clusters.** *Omics.* 2012, **16:**284-287.

8. Viborg N, Ramskov S, Andersen RS, Sturm T, Fugmann T, Bentzen AK, Rafa VM, Straten PT, Svane IM, Met Ö, Hadrup SR: **T cell recognition of novel shared breast cancer antigens is frequently observed in peripheral blood of breast cancer patients.** *Oncoimmunology.* 2019, **8:**e1663107.

9. Zach O, Lutz D: **Mammaglobin remains a useful marker for the detection of breast cancer cells in peripheral blood.** *J Clin Oncol.* 2005, **23:**3160; author reply 3160-3161.

10. Hayakawa Y, Sakitani K, Konishi M, Asfaha S, Niikura R, Tomita H, Renz BW, Tailor Y, Macchini M, Middelhoff M, et al: **Nerve Growth Factor Promotes Gastric Tumorigenesis through Aberrant Cholinergic Signaling.** *Cancer Cell.* 2017, **31:**21-34.
